# Design, Synthesis and Antifungal Activity Evaluation of New Thiazolin-4-ones as Potential Lanosterol 14α-Demethylase Inhibitors

**DOI:** 10.3390/ijms18010177

**Published:** 2017-01-17

**Authors:** Anca Stana, Dan C. Vodnar, Radu Tamaian, Adrian Pîrnău, Laurian Vlase, Ioana Ionuț, Ovidiu Oniga, Brînduşa Tiperciuc

**Affiliations:** 1 Department of Pharmaceutical Chemistry, “Iuliu Hațieganu” University of Medicine and Pharmacy, 41 Victor Babeș Street, RO-400012 Cluj-Napoca, Romania; teodora_anca@yahoo.com (A.S.); onigao65@yahoo.com (O.O.); brandu32@yahoo.com (B.T.); 2 Department of Food Science and Technology, University of Agricultural Sciences and Veterinary Medicine, 3-5 Manăştur Street, RO-400372 Cluj-Napoca, Romania; 3 National Institute for Research and Development for Cryogenic and Isotopic Technologies, 4th Uzinei Street, RO-240050 Râmnicu Vâlcea, Romania; radu.tamaian@icsi.ro; 4 SC Biotech Corp SRL, 4th Uzinei Street, RO-240050 Râmnicu Vâlcea, Romania; 5 National Institute for Research and Development of Isotopic and Molecular Technologies, RO-400293 Cluj-Napoca, Romania; adrian.pirnau@itim-cj.ro; 6 Department of Pharmaceutical Technology and Biopharmaceutics, “Iuliu Hațieganu” University of Medicine and Pharmacy, 41 Victor Babeș Street, RO-400012 Cluj-Napoca, Romania; vlaselaur@yahoo.com

**Keywords:** thiazolin-4-one, antifungal activity, lipophilicity, lanosterol 14α-demethylase, molecular docking, ADME-Tox predictors

## Abstract

Twenty-three thiazolin-4-ones were synthesized starting from phenylthioamide or thiourea derivatives by condensation with α-monochloroacetic acid or ethyl α-bromoacetate, followed by substitution in position 5 with various arylidene moieties. All the synthesized compounds were physico-chemically characterized and the IR (infrared spectra), ^1^H NMR (proton nuclear magnetic resonance), ^13^C NMR (carbon nuclear magnetic resonance) and MS (mass spectrometry) data were consistent with the assigned structures. The synthesized thiazolin-4-one derivatives were tested for antifungal properties against several strains of *Candida* and all compounds exhibited efficient anti-*Candida* activity, two of them (**9b** and **10**) being over 500-fold more active than fluconazole. Furthermore, the compounds’ lipophilicity was assessed and the compounds were subjected to in silico screening for prediction of their ADME-Tox properties (absorbtion, distribution, metabolism, excretion and toxicity). Molecular docking studies were performed to investigate the mode of action towards the fungal lanosterol 14α-demethylase, a cytochrome P450-dependent enzyme. The results of the in vitro antifungal activity screening, docking study and ADME-Tox prediction revealed that the synthesized compounds are potential anti-*Candida* agents that might act by inhibiting the fungal lanosterol 14α-demethylase and can be further optimized and developed as lead compounds.

## 1. Introduction

Of the five-membered heterocyclic rings containing sulfur and nitrogen, the thiazolin-4-ones are considered biologically privileged molecules and enjoy special attention from researchers in the field of medicinal chemistry due to their multifarious biological activities and good tolerability in humans [1,2].

Thiazolin-4-ones and their derivatives have great biological importance because they were found to have valuable pharmacological activities, such as antibacterial [1,3], antifungal [1,4], antiviral [5], anti-inflammatory (through COX inhibition) [6], hypoglycemic [7], antioxidant [8], neuroprotective and anticonvulsant [9], antitubercular [10] and antitumor [2,4,11] activities.

Despite the numerous antifungals approved for the treatment of infections, the emergence of fungal infectious diseases and the dramatically increasing number of pathogens resistant to different classes of currently available antimycotic agents resulted in the acute need to implement new strategies for developing new antifungal chemotherapeutics towards which there is little antimicrobial resistance or no cross-resistance [12]. The lanosterol 14α-demethylase (or CYP51A1) is a key enzyme in the synthesis of ergosterol, an essential component of the fungal cell membrane and constitutes an important biological target for the most popular class of antifungals (the azoles) [13]. This enzyme catalyzes the conversion of lanosterol into ergosterol through C14-demethylation. Fluconazole, a triazole derivative, and ketoconazole, an imidazole derivative, from the azole class of antifungals inhibit CYP51A1 by forming a coordinate bond between the nucleophilic nitrogen of the azole heterocycle (N3 of imidazole and N4 of triazole) and the heme iron in the ferric state from the active site of enzyme. Inhibition of lanosterol 14α-demethylase leads to accumulation of 14-a-methylsterols on the fungal surface and alteration of plasma membranes’ permeability and rigidity, which results in arrest of fungal growth. Because this enzyme is found in all eukaryotes (including humans) and because the azoles interact also with other cytochrome P450 dependent enzymes (CYP3A4), a selective inhibition towards the fungal CYP51A1 is essential for an increased therapeutic index [13]. 

The pharmacological activity of some compounds is strictly related to their lipophilicity as for the manifestation of therapeutic properties they must pass through a series of biological barriers, from the administration site to the site of action. Therefore, from the physicochemical properties of biologically active compounds, lipophilicity is of great importance and should be determined from the early stages of drug development because it affects basic steps of a drug’s pharmacokinetics and pharmacodynamics, such as absorption, distribution, metabolism, excretion and toxicity (ADME-Tox) [14].

Virtual screening (VS) is an important tool for the identification of good leads as one of the first essential steps in the drug discovery process. Therefore, the VS output allows prioritizing the development of the most promising compounds (drug-like or lead-like molecules) prior to high throughput screening (HTS) [15]. Computational prediction of lipophilicity, as a critical aspect of VS, is crucial for the improvement of ADME-Tox properties of the most promising drug/lead candidates, from the first steps. Suboptimal pharmacokinetic properties and increased toxicity are some of the most important reasons for a drug’s failure in the development phases. In silico prediction of ADME-Tox properties of new drug candidates is more economic and time sparing compared to the in vivo experimental determination of these parameters and could be useful for eliminating the molecules that are likely to fail in the early stage of drug discovery.

In order to predict the binding affinity (BA), the activity and the potential mechanism of action of a molecule, molecular docking predicts the binding orientation of small molecule drug candidates to their biological targets [16].

Based on the proven biological potential of the compounds with thiazolin-4-one core in their structure and as a continuation to our increased interest in the chemistry of thiazole and its derivatives [17], herein we report the synthesis of new thiazolin-4-ones diversely substituted in positions 2 and 5, with structures that include, in addition to the thiazolin-4-one ring, other heterocycles with known biological potential such as thiazole [18] and chromone [19]. The synthesized compounds were evaluated for their antifungal potential and, furthermore, lipophilicity studies, in silico ADME-Tox predictions and molecular docking studies were conducted in order to establish their affinity towards the biological target and a potential mechanism of action. Considering the numerous data from the literature that attest the ability to inhibit the fungal lanosterol 14α-demethylase of antifungal azoles (fluconazole, a triazole; ketoconazole, an imidazole; and ravuconazole and isavuconazole, both triazoles with a thiazole moiety in their structure) [20], thiazolidine-4-one [21] and rhodanine derivatives [22], the fungal CYP51A1 was chosen as biological target in the docking study.

## 2. Results and Discussion

### 2.1. Chemistry

A series of new 2-(allyl-amino)-5-arylidene-thiazol-4(5*H*)-ones, 2-(phenyl-amino)-5-arylidene- thiazol-4(5*H*)-ones and 2-(1-naphthyl-amino)-5-arylidene-thiazol-4(5*H*)-ones were synthesized starting from various thiourea derivatives.

**Scheme 1:**
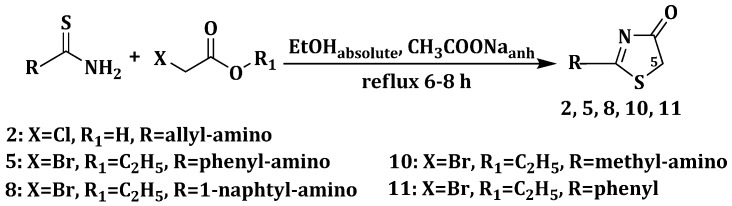
Synthesis of 2-substituted thiazolin-4-ones.

The synthetic route for the synthesis of thiazolin-4-ones, substituted in position 2 with phenyl, alkyl-amino or aryl-amino radicals is illustrated in Scheme 1. The thiazolin-4-one derivatives **2**, **5**, **8**, **10** and **11** were synthesized through the condensation of various thiourea derivatives or phenylthioamide with α-monochloroacetic acid or ethyl α-bromoacetate, in refluxing absolute ethanol, in the presence of anhydrous sodium acetate. The 2-substituted thiazolin-4-ones **2**, **5**, **8**, **10** and **11** were previously reported in the literature [23,24,25,26,27].

**Scheme 2:**
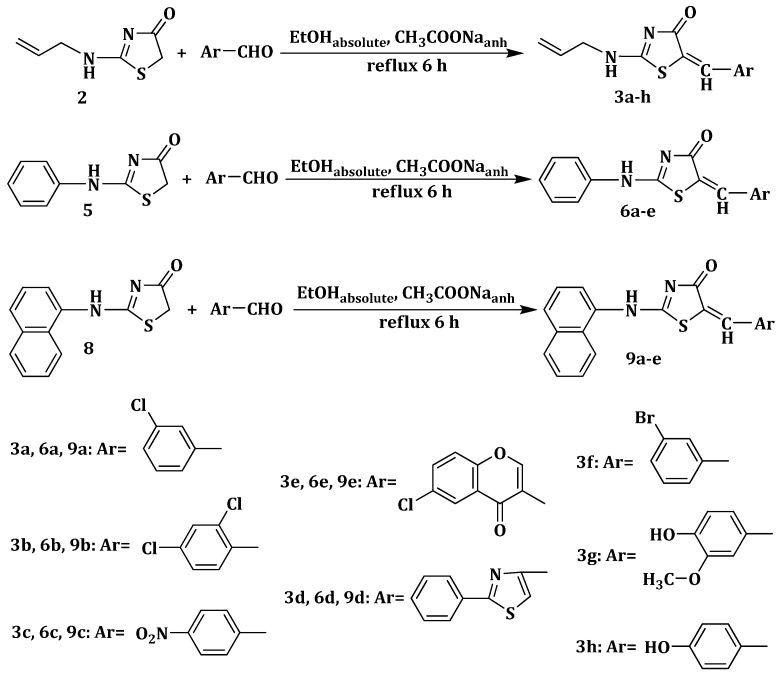
Synthesis of 5-arylidene-thiazolin-4-ones **3a**–**h**, **6a**–**e** and **9a**–**e**.

Subsequently, the position 5 of the substituted 2-amino-thiazolin-4-ones was modulated through Knoevenagel condensation of the obtained intermediates **2**, **5** and **8**, with various aromatic or heteroaromatic aldehydes, in absolute ethanol and in the presence of anhydrous sodium acetate, in order to obtain, in good yields (59%–94%) the 2-(allyl/aryl-amino)-5-arylidene-thiazolin-4-ones **3a**–**h**, **6a**–**e** and **9a**–**e** (Scheme 2).

The results of the C, H, N, S quantitative elemental analysis of the synthesized compounds were consistent with the calculated values, within ±0.4% of the theoretical values. 

The structural confirmation of the synthesized compounds was performed using spectral analysis by recording infrared spectra, mass spectra and nuclear magnetic resonance spectra. All spectral and analytical data were in accordance with the assumed structures. Details of the synthetic procedures, yields, physical, analytical and spectral data for the synthesized compounds are presented in the Experimental Section.

Mass spectra were recorded for both the 5-unsubstituted thiazolin-4-one derivatives **2**, **5**, **8**, **10**, and **11** and the 5-arylidene-thiazolin-4-ones **3a**–**h**, **6a**–**e** and **9a**–**e**, which gave information regarding the fragmentation of the compounds with their corresponding mass and, in all cases, revealed the correct molecular ion peaks (M^+^ or M ^+1^), as suggested by their molecular formulas. 

In the IR spectra of compounds **3a**–**h**, **6a**–**e** and **9a**–**e**, there is a strong absorption band at 1728–1681 cm^−1^ due to the stretching vibration of the C=O group from the thiazolin-4-one ring and a low intensity absorption band at 3438–3427 cm^−1^ due to valence vibration of the NH bond of the secondary amine. The IR spectra of 5-((6-chloro-4-oxo-4*H*-chromen-3-yl)methylene) thiazolin-4-ones **3e**, **6e** and **9e**, displayed, in addition, a high intensity absorption band at 1653–1647 cm^−1^, characteristic to νC=O stretching vibration, two strong absorption bands at 1273–1270 and 1045–1041 cm^−1^, characteristic to the stretching vibration of the C–O group and a high intensity absorption band at 1173–1167 cm^−1^, characteristic to the νC–Cl stretching vibration of the 6-chloro-4-oxo-4*H*-chromen-3-yl rest. The absorption bands at 1523–1519 and 1330–1326 cm^−1^ in the IR spectra of compounds **3c**, **6c** and **9c** are dued to the anti-symmetric and symmetric stretching vibration of the nitrogen-oxygen bonds of the aromatic NO_2_ group. The IR spectra of the compounds **3a**–**b**, **6a**–**b** and **9a**–**b** presented high intensity absorption bands at 1158–1093 cm^−1^, due to the valence vibrations of the C–Cl bonds. Compound **3g** exhibited in the IR spectrum two strong absorption bands at 1046 and 1019 cm^−1^ due to the νC–O stretching vibration of the phenyl-methyl-ether group. In the IR spectra of compounds **3g** and **3h**, the phenolic OH group registered an absorption band at 1336–1331 cm^−1^ corresponding to the νC–O valence vibration and an absorption band at 3289–3285 cm^−1^ corresponding to the νO–H stretching vibration.

In the ^1^H NMR spectra of compounds **3a**–**h**, **6a**–**e** and **9a**–**h** were recorded the signal characteristic for the methine proton (=C**H**–) as a singlet in the 7.50–7.74 ppm region, which confirmed the success of the Knoevenagel condensation between the 2-amino substituted thiazolin-4-ones and the corresponding aromatic aldehydes, with the formation of 2-(aryl/allyl-amino)-5-arylidene-thiazolin-4-ones. The absence of a singlet signal, corresponding to the methylene protons from the 5th position of the thiazolin-4-one core, at 3.97–4.12 ppm in the ^1^H NMR spectra of all the newly synthesized compounds, further confirmed the formation of the 5-substituted derivatives. In the 7.04–8.91 ppm region of the ^1^H NMR spectra, the aromatic protons resonated as characteristic doublets, triplets and multiplets, that appeared as split signals based on the presence of other protons in their immediate vicinity. The phenolic OH group proton appeared as a singlet at 9.31–9.35 ppm. The protons of the methoxy group resonated as a singlet in the 3.83 ppm region of the spectrum. The ^13^C NMR spectra of the synthesized compounds were consistent with the proposed structures.

### 2.2. Antifungal Activity

#### 2.2.1. Determination of Inhibition Zone Diameters

All synthesized compounds were initially subjected to in vitro antifungal screening using the cup-plate agar diffusion method, against a fungal strain of *Candida albicans* ATCC 10231.

Fluconazole is an antifungal triazole that belongs to the well represented azole class and it is currently used to treat a wide variety of fungal infections. It acts by inhibiting the fungal lanosterol 14α-demethylase, its mechanism of action involving the nucleophilic nitrogen of the azole heterocycle coordinating as the sixth ligand of the heme iron in the ferric state of the enzyme [13]. It was chosen as positive control due to the presence of two triazole rings in its structure that are essential for the biological activity.

The results of the antimicrobial activity testing of the 2-substituted-thiazolin-4-ones **2**, **5**, **8**, **10**, and **11** (1 mg/mL), and of the 2-(allyl/aryl-amino)-5-arylidene-thiazolin-4-ones **3a**–**h**, **6a**–**e** and **9a**–**e** (1 mg/mL) in comparison with those of the reference compound, fluconazole (1 mg/mL), are given in Table 1.

All the synthesized compounds showed moderate to good inhibitory activity against *C. albicans* ATCC 10231 (16–22 mm inhibition zone diameters) (Table 1). Of these, compounds **5**, **3f**, **3g**, **8**, **9e** and **10** exhibited similar or better antifungal activities than that of fluconazole, used as reference antimycotic (*p* < 0.05). The 5-unsubstituted 2-(alkyl/aryl-amino)-thiazolin-4-ones **5**, **8**, and **10** and the 2-(allylamino)-5-arylidene-thiazolin-4-ones **3a**–**h** were generally more active than the rest of the compounds against *C. albicans* ATCC 10231 at tested concentration, suggesting that the presence of an allyl substituent at the exocyclic amine from position 2 of the thiazolin-4-onic core is favorable to the antifungal activity.

#### 2.2.2. Determination of Minimum Inhibitory Concentration (MIC) and Minimum Fungicidal Concentration (MFC) Values

The incidence of fungal infections has increased significantly over the past decades, thus contributing to morbidity and mortality through microbial infections. Candida species are the major human fungal pathogens that cause both mucosal and deep tissue infections and over 90% of invasive infections are caused by *Candida albicans*, *Candida glabrata*, *Candida parapsilosis*, *Candida tropicalis* and *Candida krusei* [28].

Prompted by the results obtained in the antimicrobial screening using the agar diffusion method, minimum inhibitory concentrations and fungicidal concentrations were determined, employing the broth microdilution method. All the synthesized compounds were tested against four strains of fungi (*Candida albicans* ATCC 10231, *Candida albicans* ATCC 18804, *Candida krusei* ATCC 6258 and *Candida parapsilosis* ATCC 22019). As reference antifungals, fluconazole (a systemic use azole, also used as positive control in the previous study) and ketoconazole (a topical azole) were chosen. 

The results of the minimum inhibitory concentration test and those of the minimum fungicidal concentration assay are depicted in Table 2.

The antifungal activity against two strains of *C. albicans*, one *C. krusei* strain and one strain of *C. parapsilosis* showed MIC values ranging from 0.015 µg/mL (compounds **10** and **9b**) to 31.25 µg/mL and MFC values ranging from 0.015 µg/mL (compounds **10** and **9b**) to 62.5 µg/mL. Most of the compounds exhibited similar or much higher MIC and MFC values than those of fluconazole (MIC = 7.81–15.62 µg/mL, MFC = 15.62–31.25 µg/mL) and ketoconazole (MIC = 3.9–7.81 µg/mL, MFC = 7.81–15.62 µg/mL). All the compounds displayed similar or much better antifungal activity than fluconazole (*p* << 0.05), and of these, 16 were more active than ketoconazole (*p* << 0.05) against *C. krusei* ATCC 6258. In addition, 16 compounds presented better inhibitory activity than fluconazole (*p* << 0.05) and 15 displayed better antifungal potential than ketoconazole (*p* << 0.05) against the *C. albicans* strains used in the assay. All the compounds except **3c** exhibited similar or much higher MIC and MFC values against *C. parapsilosis* ATCC 22019 than fluconazole (*p* << 0.05) and of these, 18 were as active as or even more active than ketoconazole (*p* << 0.05).

Overall, the synthesized thiazolin-4-ones presented good to excellent antifungal activities. The MFC/MIC ratio for all tested compounds ranged from 1 to 4, suggesting that the synthesized thiazolin-4-one derivatives could act as fungicidal agents [29]. The most active compounds were the 2-(methylamino)thiazol-4(5*H*)-one **10** and the 5-(2,4-dichlorobenzylidene)-2-(naphthalen-1-ylamino) thiazol-4(5*H*)-one **9b**, being over 250 times more active than ketoconazole.

### 2.3. Lipophilicity Evaluation

The lipophilicity of the synthesized compounds was assessed using PCA based on RP-TLC data. Compounds **3f**, **10** and **11** were not included in this study because their *R*_f_ could not be calculated due to the lack of their spots’ visibility in UV light.

In Table 3 are presented from the largest to the smallest, the eigenvalues of the covariance matrix, the proportion and also the difference between each eigenvalue and the next smallest one. The obtained results suggested a one component model as the first principal component (*P*_1_) explained 96.609% of the total initial information (variance). 

For a better interpretation and a better graphical representation of the obtained results, we took into consideration the scores of the first two principal components obtained for the tested compounds. The eigenvectors associated with the first two principal components are displayed in Table 4. The results obtained confirmed that the five mobile phases used are strongly related to each other so they could be reduced. This shows that for all the investigated compounds there was a regular increase in *R*_f_ values with the increasing concentration of the organic modifier (*i*-propanol).

The results of the regression analysis are compiled in Table 5. The statistics obtained are in accordance with the experimental data, the linear model explaining approximately 95% of the total variance (see *R*^2^ values) in most of the cases.

A good correlation (*R*^2^ = 0.9507) was found between the intercept’s—*R*_M0_ (lipophilicity)—and the slope’s—b (specific hydrophobic surface area)—values (Table 5). This suggested that the compounds used in this study might form a homologous series. In addition, a very good correlation (*R*^2^ = 0.901) was observed between *R*_M0_ and the scores of the first principal component (*P*_1_) as described by the following equation (Equation (1)):

*R*_M0_ = 1.9864 − 0.3317 × *P*_1_ (1)

**Figure 1:**
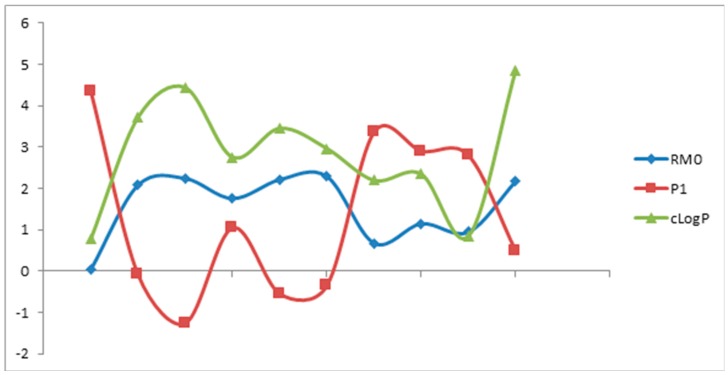
The relationship between experimental lipophilicity indices (*R*_M0_, *P*_1_) and computed cLogP values.

**Figure 2:**
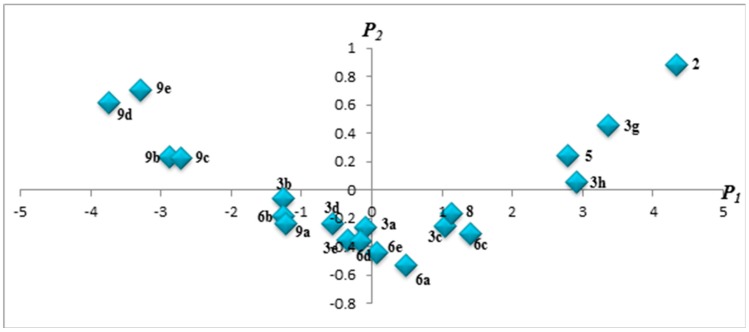
Congeneric lipophilicity chart (2D plot) obtained by plotting the scores corresponding to *P*_1_ and *P*_2_.

Based on these findings and as can be seen in data provided in Table 5, the scores of the first principal component can efficiently replace the *R*_M0_ values in the estimation experiments of these compounds’ lipophilicity. It can be observed that *R*_M0_ and *P*_1_ profiles (Figure 1) are well correlated with the profile of the computed cLogP values, all describing similar patterns. Furthermore, scores plots are very useful as a display tool for observing the relationship between compounds, looking for trends, groupings or outliers, as shown in Figure 2. Therefore, by graphing scores onto the plane described by the first two principal components, *P*_1_ and *P*_2_, a “congeneric lipophilicity chart” was obtained, where a highly lipophilic compound was characterized by a low value of the *P*_1_ parameter.

The highest values of the lipophilicity parameters were obtained for compounds **9d**, **9e**, **9b**, **9c**, **3b**, **6b** and **9a** while the lowest values were characteristic for compounds **2**, **3g**, **3h**, **5**, **6c**, **8** and **3c**. By analyzing the relationships between the compounds’ chemical structures and *P*_1_ values as a parameter used for estimating lipophilicity it was observed that the lipophilic character of the compounds was significantly influenced by both substituents from position 2 and 5 of the thiazolin-4-one ring. Regarding the 5-unsubstituted thiazolin-4-one derivatives, the substituent in position 2 caused an increase in lipophilicity with the increase of its molecular mass; thereby the most lipophilic 5-unsubstituted thiazolin-4-one derivatives were the 2-α-naphthylamino- thiazolin-4-one **8**, followed by the less lipophilic 2-phenylamino-thiazolin-4-one **5** and then by the least lipophilic 2-allylamino-thiazolin-4-one **2**.

For all the studied compounds it was observed that the substitution in position 5 of the thiazolin-4-onic heterocycle led to an increase in lipophilicity.

The lowest lipophilicity was registered for the compounds with an allylamino rest in position 2 of the thiazolin-4-one moiety. By introducing an arylidene or hetarylidene rest in position 5, a lipophilicity augmentation was noticed. The presence of a polar group (OH, OCH_3_) on the phenyl rest from position 5 determined a less pronounced lipophilicity raise. Moreover, the introduction of one or two chloride atoms on the aromatic or heteroaromatic ring generally resulted in an increase of the compound’s lipophilicity. 

### 2.4. Virtual Screening 

All thiazolin-4-one derivatives and the reference compounds (fluconazole (Flu), and ketoconazole (Ket)) were virtually screened for prediction of the ADME-Tox properties and docked against fungal lanosterol 14α-demethylase, the biological target of the antifungals used as positive control in the previous studies. 

#### 2.4.1. ADME-Tox Predictions

The results of VS carried out with FAF-Drugs3 [30] are presented subsequently for lead-likeness, drug-likeness descriptors and penetration of the blood–brain barrier (Table 6). Additional data are presented in Table S1, referring to protein–protein interactions (PPIs), undesirable moieties and substructures involved (UMSs) in toxicity problems, respectively in Table S2 referring to covalent inhibitors and PAINS (Pan Assay Interference Compounds) moieties. In Table S3 are presented the results of VS using the customized filters developed by well-known pharmaceutical companies for drug safety profiling.

The results of VS, carried out with FAF-Drugs3, are summarized in Table 6. These were carried out for the following lead-likeness and drug-likeness descriptors: molecular weight (MW, expressed in Daltons), the logarithm of the partition coefficient between *n*-octanol and water (LogP) characterizing lipophilicity, hydrogen bond donors (H_BD_, sum of all –OHs and –NHs, according to Lipinski’s rule of five (RO5) definition [31]), hydrogen bond acceptors (H_BA_, sum of all oxygen and nitrogen atoms, according to RO5 definition [31]), topological Polar Surface Area (tPSA, summation of tabulated surface contributions of polar fragments—topologically calculated), number of rotatable bonds (RtB, number of any single non-ring bond, bounded to non-terminal heavy atom—amide C–N bonds are not considered because of their high rotational energy barrier [32]), number of rigid bonds (RiB, number of non-flexible bonds—e.g., double and triple bonds, bonds involved in ring systems and additionally amide bonds), number of the smallest set of smallest rings (R_s_, the smallest ring building blocks necessary to form other ring systems), maximum size of the biggest ring system (M_x_S, number of atoms involved in the biggest ring system), number of carbon atoms (C_s_), number of heteroatoms (HA, number of non-carbon atoms—hydrogen atoms are not included), the ratio between the number of non-carbon atoms and the number of carbon atoms (H/C), number of charged groups (Crg), formal total charge of the compound (T_Crg_) and stereo centers (SC—computed only for leads, the presence of stereo centers and the number of chiral centers, are descriptors of complexity). The activity at the central nervous system level (CNS), respectively the penetration of blood–brain barrier (BBB) were evaluated only based on the values predicted for MW, LogP, H_BA_, H_BD_ and tPSA, as specified in the literature [33].

As it can be observed in Table 6, more than half of the synthesized thiazolin-4-one derivatives (**2**, **3a**, **3c**–**h**, **5**, **6c**, **8** and **11**) were qualified as potential leads. Only one thiazolin-4-one derivative (**10**) cannot be qualified as a good lead due its way too low MW, meanwhile the rest of rejected thiazolin-4-one derivatives exhibited higher values than upper thresholds for LogP (**3b, 6a**, **6b**, **6d**, **6e**, and **9a**–**e**). Except **9b**, all thiazolin-4-one derivatives were accepted as drug-like compounds. In addition, the predictions made for **9b** and four of the drug-like thiazolin-4-one derivatives (**3c**, **6c**, **9c** and **10**) indicated their inability to penetrate the BBB. Therefore, for those compounds, a CNS level inactivity is forecasted. Additionally, from Table S1 it can be observed that all thiazolin-4-one derivatives comply with Veber’s rule [32] and Egan’s rule [34] regarding the molecular properties that influence oral bioavailability. In addition, all thiazolin-4-one derivatives fit into RO5 for oral bioavailability [31], even though the computations for five compounds (**6b**, **9a**, **9b**, **9d** and **9e**) slightly overpassed the threshold values for LogP considered by Christopher A. Lipinski and coworkers [31], this being only one violation of the rule, while the predictions for their oral bioavailability are considered good.

Table S1 summarizes the results of VS carried out with FAF-Drugs3 for detection of non-peptidic inhibitors of PPI and UMSs involved in toxicity problems. It can be observed that only eight thiazolin-4-one derivatives (**6c**–**e**, and **9a**–**e**) are PPI friendly, same as **Ket**, the rest of compounds requiring further structural optimization prior to their use as possible “hits” in the drug development process. Moreover, those PPI friendly derivatives and three compounds which require further structural optimization (**2**, **6a**–**b**) should be refined in order to decrease even more their toxicity predictions generated by the presence of Michael acceptors (double bonds)—a “warhead” moiety present in the structure of all those compounds, together with other low risk UMSs: terminal vinyl from the structure of derivative **2**, thiazole found in **6d** and **9d**, respectively nitro and nitrobenzene present in **6c** and **9c**. In the case of eight 2-(allyl-amino)-5-arylidene-thiazolin-4-ones (**3a**–**h**) the prediction is bad, the compounds being rejected by the imposed filter, due the presence of high risk Michael acceptors in their structure combined with some low risk moieties: terminal vinyl (detected also in all of those derivatives), nitro and nitrobenzene (found in **3c**), thiazole (found in **3d**), and phenol (found in the structure of **3g** and **3h**). Four of the not so PPI friendly derivatives were found free of UMSs: **5**, **8**, **10** and **11** (all are 5-unsubstituted thiazolin-4-one derivatives).

Table S2 summarizes the results of VS carried out with FAF-Drugs3, in order to detect the problematic groups involved in covalent binding with biological macromolecules and structural alerts for PAINS, compounds that appear as frequent hitters in HTS. The results presented in Table S2 indicated that the same UMSs-free 5-unsubstituted thiazolin-4-one derivatives, as shown in Table S1 (**5**, **8**, **10** and **11**), do not contain any covalent inhibitor group—the reference compounds are all free of this kind of substructures. The rest of the thiazolin-4-one derivatives have Michael acceptors (double bonds) in their structure, functional groups responsible for both electrophilic protein-reactive false positives in HTS [35,36] and covalent binding with peptides. Moreover, with the exception of derivative **2**, the rest of thiazolin-4-one derivatives contain a second covalent inhibitor: α, β-unsaturated carbonyl, substructure with possible undesirable effects [37]. According to two PAINS filters (A and C), all 5-unsubstituted thiazolin-4-one derivatives are free of “promiscuous substructures”, meanwhile the filter B detected a low-risk structural alert at compounds **3a**–**h**, **6a**–**e** and **9a**–**e**.

Table S3 shows the results of VS using the customized filters for drug safety profiling: the GSK 4/400 rule, the Pfizer 3/75 rule, phospholipidosis induction and the MedChem rules (for identifying potentially reactive or promiscuous compounds) [35]. 

The integrated analysis of various rules taken into consideration for drug safety profiling (Table S3) indicates possible safety issues at the administration of **9d**–**e**, according to GSK 4/400 rule (this rule is based on lowering the MW and LogP area of physicochemical property space to obtain improved ADME-Tox parameters), the rest of the screened compounds having a good prediction. On the other hand, according to the Pfizer 3/75 rule, compounds **3a**–**b**, **3f**, **6a**–**b** are likely to cause toxicity and experimental promiscuity, because they have LogP > 3 and tPSA < 75 Å^2^ (as shown in Table 6). 

None of the thiazolin-4-one derivatives were classified as phospholipidosis inducers, meanwhile **Ket** is considered as being one. 

Compound **11** successfully overpassed the thresholds of MedChem rules; meanwhile the rest of thiazolin-4-one derivatives and the reference compound (**Ket**) may exhibit some safety issues due to the presence of various flagged substructures. A flagged interference, the amino_naphthalene rule, is related to posible lack of druggability attendant on excessive lipophilicity and flexibility due to the number of successive methylenes (C4 through C7) detected in compounds **8** and **9a**–**e**. Moreover, some problematic substructures were also detected in one of the reference compounds (**Ket**), but, according to the MedChem rules, 19 drugs out of 123 best-selling drugs failed the rules as an outright rejection, and another 18 failed on demerits. 

#### 2.4.2. Docking against the Fungal Lanosterol-14-α-demethylase 

Lanosterol 14-α demethylase is a cytochrome P450 enzyme that plays a crucial role in sterol biosynthesis in eukaryotes because it catalyzes the C14-demethylation of lanosterol which is critical for ergosterol biosynthesis, a fundamental component of fungal membranes. The azole antifungal drugs selectively inhibit the fungal lanosterol 14α-demethylase, their mechanism of action involving the nucleophilic nitrogen of the azole heterocycle coordinating as the sixth ligand of the heme iron in the ferric state and the azole drug side chains interacting with the polypeptide structure [13].

All investigated molecules (the thiazolin-4-one derivatives and the reference compounds) were virtually subjected to docking, against the designated fungal target (lanosterol 14-α demethylase) in order to investigate the potential binding mode and binding affinity of these inhibitors. Docked ligand conformations (poses) were analyzed in terms of binding affinity (BA, expressed in kcal/mol) and hydrogen bonding between the best poses and their target protein. Detailed analyses of the ligand–receptor interactions were carried out, and final possible orientations of the ligands and receptors were saved. 

**Figure 3:**
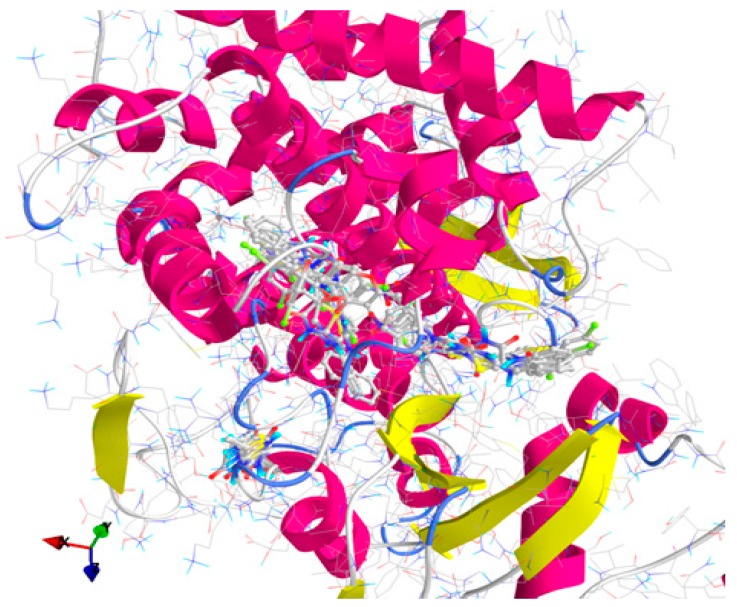
General view of docking poses of the screened compounds in the active site of lanosterol-14α-demethylase (target is depicted as thin sticks with secondary structure drawn as cartoon backbone, meanwhile ligands are figured as sticks): there can be described three binding patterns—Group A (**left**): **2**, **3a**, **3c**, **3f**–**h**, **5**, **8**, **10** and **11**; Group B (**right**): **3b**, **3d**–**e**, **6d**, **9a**–**9c** and **Ket**; and Group C (**center**): **6a**–**c**, **6e** and **Flu**.

All thiazolin-4-one derivatives and the antifungal reference compounds (**Flu** and **Ket**) were docked against the generated homology model for lanosterol-14α-demethylase and the results are presented in Table 7 and Figure 3 and Figure S1.

The results of docking against lanosterol-14α-demethylase (Table 7) indicated that most of the screened derivatives and **Flu** make H-bonds especially with amino acids residues with polar uncharged side chains (asparagine, serine and threonine), meanwhile 10 derivatives (**3b**, **3d**, **6b**–**e** and **9c**–**e**) and **Ket** do not interact with their target by hydrogen bonding. Three derivatives (**3e**, **5** and **6a**) bind to amino acids residues with hydrophobic side chains and only one compound (**9b**) binds to histidine, an amino acid residue with an electropositively charged side chain. Moreover, compound **5** binds both to amino acids residues with polar uncharged side chains (Ser436) and hydrophobic side chains (Tyr460). Some of the tested derivatives (**2**, **10** and **11**) are weaker inhibitors than **Flu** (the weakest inhibitor from the two reference compounds), meanwhile compounds **6d**, **6e** and **9a**–**e** are stronger inhibitors than **Ket**.

## 3. Materials and Methods

### 3.1. Chemistry

All chemicals (reagent grade) were obtained from commercial sources and were used as supplied, without further purification.

Melting points were determined with an MPM-H1 Electrothermal melting point meter using the open glass capillary method and are uncorrected. The reaction progress and purity of the synthesized compounds were monitored by analytical thin layer chromatography (TLC) using Merck precoated Silica Gel 60F_254_ sheets (Darmstadt, Germany), heptane–ethyl-acetate 3:7 elution system and UV light for visualization. Elemental analysis was registered with a Vario El CHNS instrument (Hanau, Germany) and the results obtained for all synthesized compounds were in agreement with the calculated values within ±0.4% range. MS analyses were performed at 70 eV with an Agilent gas chromatograph 6890 (Darmstadt, Germany) equipped with an apolar Macherey Nagel Permabond SE 52 capillary column (Dueren, Germany) and with an LC-MS Shimadzu Mass Spectrometer (Shimadzu Corporation, Torrance, CA, USA). IR spectra were recorded using the ATR technique (Attenuated Total Reflectance) on a JASCO FT-IR—4100 spectrometer (Cremella, Italy). Nuclear magnetic resonance (^1^H NMR) spectra were recorded on a Bruker Avance NMR spectrometer (Karlsruhe, Germany) operating at 400 and 500 MHz, at room temperature, using tetramethylsilane (TMS) as internal standard. Chemical shifts were reported in ppm (δ). The samples were prepared by dissolving the compounds in DMSO-*d*_6_ (δ_H_ = 2.51 ppm) as solvent and the spectra were recorded using a single excitation pulse of 12 μs (^1^H NMR). Spin multiplets are given as *s* (singlet), *d* (doublet), *t* (triplet) and *m* (multiplet). ^13^C NMR spectra were recorded on Bruker Avance NMR spectrometer (Karlsruhe, Germany) operating at 125 MHz in DMSO-*d*_6_, using a waltz-16 decoupling scheme. All spectral analyses data were in accordance with the assigned structures.

#### 3.1.1. General Procedure for the Synthesis of 2-Substituted Thiazol-4(5*H*)-ones **2**, **5**, **8**, **10** and **11**

To a solution of 20 mmol of the appropriate thiourea derivative or phenylthioamide in absolute ethanol (15 mL) were added 40 mmol (3.28 g) of anhydrous sodium acetate and 20 mmol ethyl α-bromoacetate or α-monochloroacetic acid. The obtained reaction mixture was refluxed for 6–8 h and, after cooling, it was poured into ice-cold water. Subsequently, the resulted precipitate was isolated by filtration and washed on filter with a mixture of ethanol and distilled water. Afterwards, the crude product was purified by recrystallization from absolute ethanol in order to obtain the pure 2-substituted thiazol-4(5*H*)-one.

Compounds **2**, **5**, **8**, **10** and **11** were previously reported in the literature [23,24,25,26,27] and were synthesized following the procedure described above.

*2-(allylamino)thiazol-4(5H)-one* **2**: yield 60%, m.p. 90–91 °C (lit [23], m.p. 89–90 °C).
*2-(phenylamino)thiazol-4(5H)-one* **5**: yield 75%, m.p. 179 °C (lit [24], m.p. 178–179 °C).
*2-(naphthalen-1-ylamino)thiazol-4(5H)-one* **8**: yield 89%, m.p. 213 °C (lit [25], m.p. 213–214 °C).
*2-(methylamino)thiazol-4(5H)-one* **10**: yield 78%, m.p. 196–197 °C (lit [26], m.p. 196–199 °C).
*2-phenylthiazol-4(5H)-one* **11**: yield 59%, m.p. 108–110 °C (lit [27], m.p. 107 °C).

#### 3.1.2. General Procedure for the Synthesis of 2-(Allyl/aryl-amino)-5-arylidene-thiazolin-4-ones **3a**–**h**, **6a**–**e** and **9a**–**e**

2 mmol of the appropriate 2-(allyl/aryl-amino)-thiazolin-4-one were suspended in 8 mL of absolute ethanol and then, to the obtained suspension were added 8 mmol (0.656 g) of anhydrous sodium acetate and 2 mmol of the corresponding aromatic aldehyde. The reaction mixture was refluxed for 6 h, and, after the reaction was completed, it was dropwise poured into ice-cold water. The resulting precipitate was filtered off and washed on the filter with distilled water and ethanol. The obtained compound was recrystallized from an appropriate solvent (absolute ethanol or absolute methanol).

*2-(Allylamino)-5-(3-chlorobenzylidene)thiazol-4(5H)-one* (**3a**). Yield 87% (0.485 g); light yellow powder; m.p. 198 °C; IR (ATR, ν (cm^−1^)): 3437 (N–H_amine_), 1726 (C=O_TZ_), 1155 (C–Cl); ^1^H NMR (500 MHz, DMSO-*d_6_*, δ/ppm): 7.74 (t, 1H, NH), 7.50 (s, 1H, –CH=), 7.36–7.50 (m, 4H, Ar–H), 6.03 (m, 1H, CH=), 5.23 (d, 2H, =CH_2_), 3.98 (m, 2H, CH_2_); ^13^C NMR (125 MHz, DMSO-*d*_6_, δ/ppm): 168.12 (C=O), 157.23 (C), 139.54 (CH), 136.75 (C), 135.62 (C), 133.81 (C), 131.05 (CH), 130.55 (CH), 129.11 (CH), 128.33 (CH), 126.27 (CH), 118.44 (CH_2_), 50.15 (CH_2_); MS (EI, 70 eV) *m*/*z* (%): 279 (M + 1, 100), 280 (M + 2, 10.6), 281 (M + 3, 36.8), 252 (29.0), 226 (6.7), 209 (22.6), 195 (100), 176 (19.0); Anal. Calcd. for C_13_H_11_ClN_2_OS (278.76): C, 56.01; H, 3.98; N, 10.05; S, 11.50; Found: C, 56.32; H, 3.79; N, 10.12; S, 11.52.

*2-(Allylamino)-5-(2,4-dichlorobenzylidene)thiazol-4(5H)-one* (**3b**). Yield 65% (0.407 g); white powder; m.p. 238 °C; IR (ATR, ν (cm^−1^)): 3438 (N–H_amine_), 1714 (C=O_TZ_), 1158 (C–Cl), 1093 (C–Cl); ^1^H NMR (500 MHz, DMSO-*d*_6_, δ/ppm): 7.93 (t, 1H, NH), 7.56 (s, 1H, –CH=), 7.10–7.12 (m, 3H, Ar–H), 6.31 (m, 1H, CH=), 5.34 (d, 2H, =CH_2_), 3.66 (m, 2H, CH_2_); ^13^C NMR (125 MHz, DMSO-*d*_6_, δ/ppm): 166.20 (C=O), 158.42 (C), 138.94 (CH), 137.77 (C), 135.31 (C), 133.15 (C), 131.56 (C), 130.99 (CH), 129.78 (CH), 126.63 (CH), 124.88 (CH), 117.08 (CH_2_), 49.94 (CH_2_); MS (EI, 70 eV) *m*/*z* (%): 313 (M + 1, 100), 314 (M + 2, 13.3), 315 (M + 3, 95.5), 316 (M + 4, 17.3), 317 (M + 5, 19.2), 277 (100), 256 (7.2), 208 (14.1), 185 (5.9), 149 (5.1); Anal. Calcd. for C_13_H_10_Cl_2_N_2_OS (313.20): C, 49.85; H, 3.22; N, 8.94; S, 10.24; Found: C, 49.93; H, 3.35; N, 8.98; S, 10.36.

*2-(Allylamino)-5-(4-nitrobenzylidene)thiazol-4(5H)-one* (**3c**). Yield 89% (0.514 g); pale yellow powder; m.p. 265 °C; IR (ATR, ν (cm^−1^)): 3431 (N–H_amine_), 1705 (C=O_TZ_), 1523 (NO_2_ asymmetric), 1326 (NO_2_ symmetric); ^1^H NMR (500 MHz, DMSO-*d*_6_, δ/ppm): 7.98 (t, 1H, NH), 7.62 (s, 1H, –CH=), 7.13–7.84 (m, 4H, Ar–H), 5.98 (m, 1H, CH=), 5.25 (d, 2H, =CH_2_), 3.84 (m, 2H, CH_2_); ^13^C NMR (125 MHz, DMSO-*d*_6_, δ/ppm): 175.30 (C=O), 161.57 (C), 148.11 (C), 142.59 (C), 138.72 (CH), 134.09 (C), 131.25 (2CH), 129.44 (CH), 125.22 (2CH), 117.45 (CH_2_), 50.11 (CH_2_); MS (EI, 70 eV) *m*/*z* (%): 290 (M + 1, 100), 291 (M + 2, 17.2), 292 (M + 3, 6.3), 248 (97.0), 245 (100), 203 (7.3), 174 (10.2), 141 (17.9); Anal. Calcd. for C_13_H_11_N_3_O_3_S (289.31): C, 53.97; H, 3.83; N, 14.52; S, 11.08; Found: C, 53.88; H, 3.97; N, 14.67; S, 11.23.

*2-(Allylamino)-5-((2-phenylthiazol-4-yl)methylene)thiazol-4(5H)-one* (**3d**). Yield 80% (0.523 g); white powder; m.p. 339 °C; IR (ATR, ν (cm^−1^)): 3435 (N–H_amine_), 1728 (C=O_TZ_); ^1^H NMR (500 MHz, DMSO-*d*_6_, δ/ppm): 8.02 (s, 1H, NH), 8.00 (s, 1H, C5-thiazole-H), 7.70 (s, 1H, –CH=), 7.42–7.71 (m, 5H, Ar–H), 6.05 (m, 1H, CH=), 5.32 (d, 2H, =CH_2_), 4.15 (m, 2H, CH_2_); ^13^C NMR (125 MHz, DMSO-*d*_6_, δ/ppm): 172.20 (C=O), 169.85 (C), 158.33 (C), 150.08 (C), 140.88 (CH), 138.63 (C), 134.66 (CH), 133.19 (C), 130.97 (2CH), 129.14 (2CH), 128.93 (CH), 127.51 (CH), 117.68 (CH_2_), 50.42 (CH_2_); MS (EI, 70 eV) *m*/*z* (%): 328 (M + 1, 100), 329 (M + 2, 11.7), 330 (M + 3, 11.5), 301 (5.1), 287 (28.7), 261 (22.5), 246 (39.6), 218 (100), 155 (14.1); Anal. Calcd. for C_16_H_13_N_3_OS_2_ (327.42): C, 58.69; H, 4.00; N, 12.83; S, 19.59; Found: C, 58.82; H, 4.21; N, 12.99; S, 19.68.

*2-(Allylamino)-5-((6-chloro-4-oxo-4H-chromen-3-yl)methylene)thiazol-4(5H)-one* (**3e**). Yield 91% (0.631 g); pale pink powder; m.p. 299–301 °C; IR (ATR, ν (cm^−1^)): 3432 (N–H_amine_), 1684 (C=O_TZ_), 1647 (C=O_chromone_), 1273 (C–O_chromone_), 1167 (C–Cl), 1045 (C–O_chromone_); ^1^H NMR (500 MHz, DMSO-*d*_6_, δ/ppm): 7.93 (t, 1H, NH), 7.74 (s, 1H, –CH=), 7.11–8.74 (m, 4H, Ar–H), 6.27 (m, 1H, CH=), 5.27 (d, 2H, =CH_2_), 3.73 (m, 2H, CH_2_); ^13^C NMR (125 MHz, DMSO-*d*_6_, δ/ppm): 178.55 (C=O), 167.64 (C=O), 159.21 (C), 155.82 (C), 153.03 (CH), 149.43 (CH), 136.97 (C), 136.16 (CH), 134.68 (CH), 130.74 (CH), 128.42 (C), 125.55 (C), 119.25 (C), 119.01 (CH), 118.14 (CH_2_), 51.26 (CH_2_); MS (EI, 70 eV) *m*/*z* (%): 347 (M + 1, 100), 348 (M + 2, 19.9), 349 (M + 3, 33.8), 330 (9.8), 305 (36.5), 290 (14.8), 277 (20.3), 265 (74.2), 237 (100), 213 (1.9), 205 (1.8), 155 (26.7); Anal. Calcd. for C_16_H_11_ClN_2_O_3_S (346.79): C, 55.41; H, 3.20; N, 8.08; S, 9.25; Found: C, 55.65; H, 3.33; N, 8.31; S, 9.34.

*2-(Allylamino)-5-(3-bromobenzylidene)thiazol-4(5H)-one* (**3f**). Yield 75% (0.485 g); white powder; m.p. 330 °C; IR (ATR, ν (cm^−1^)): 3429 (N–H_amine_), 1689 (C=O_TZ_),1035 (C–Br); ^1^H NMR (500 MHz, DMSO-*d*_6_, δ/ppm): 7.96 (t, 1H, NH), 7.58 (s, 1H, –CH=), 7.44–7.86 (m, 4H, Ar–H), 6.30 (m, 1H, CH=), 5.29 (d, 2H, =CH_2_), 3.86 (m, 2H, CH_2_); ^13^C NMR (125 MHz, DMSO-*d*_6_, δ/ppm): 168.74 (C=O), 158.62 (C), 138.42 (CH), 136.33 (C), 135.10 (C), 133.96 (C), 131.14 (CH), 130.27 (CH), 129.19 (CH), 128.45 (CH), 126.47 (CH), 118.21 (CH_2_), 51.17 (CH_2_); MS (EI, 70 eV) *m*/*z* (%): 323 (M + 1, 100), 324 (M + 2, 52.1), 325 (M + 3, 50.1), 306 (9.0), 279 (17.6), 259 (13.6), 240 (20.8), 235 (11.0), 177 (17.7), 161 (22.6), 145 (9.3), 121 (12.7); Anal. Calcd. for C_13_H_11_BrN_2_OS (323.21): C, 48.31; H, 3.43; N, 8.67; S, 9.92; Found: C, 48.45; H, 3.55; N, 8.54; S, 9.85.

*2-(Allylamino)-5-(4-hydroxy-3-methoxybenzylidene)thiazol-4(5H)-one* (**3g**). Yield 77% (0.447 g); pale yellow powder; m.p. 240 °C; IR (ATR, ν (cm^−1^)): 3435 (N–H_amine_), 3285 (O–H_phenol_), 1705 (C=O_TZ_), 1336 (C–O_phenol_), 1046 (C–O_methoxy_), 1019 (C–O_methoxy_); ^1^H NMR (500 MHz, DMSO-*d*_6_, δ/ppm): 9.31 (s, 1H, OH), 8.06 (t, 1H, NH), 7.72 (s, 1H, –CH=), 7.13–7.74 (m, 3H, Ar–H), 6.31 (m, 1H, CH=), 5.34 (d, 2H, =CH_2_), 4.01 (m, 2H, CH_2_), 3.83 (s, 3H, –OCH_3_); ^13^C NMR (125 MHz, DMSO-*d*_6_, δ/ppm): 187.08 (C=O), 174.77 (C), 159.42 (C), 152.06 (CH), 148.20 (C), 134.12 (CH), 132.66 (C), 129.01 (C), 122.81 (CH), 117.30 (CH_2_), 116.57 (CH), 111.96 (CH), 55.13 (CH_3_), 50.56 (CH_2_); MS (EI, 70 eV) *m*/*z* (%): 291 (M + 1, 100), 292 (M + 2, 12.8), 293 (M + 3, 13.2), 265 (5.1), 238 (3.1), 180 (6.6), 138 (10.6); Anal. Calcd. for C_14_H_14_N_2_O_3_S (290.34): C, 57.92; H, 4.86; N, 9.65; S, 11.04; Found: C, 57.87; H, 4.83; N, 9.61; S, 11.17.

*2-(Allylamino)-5-(4-hydroxybenzylidene)thiazol-4(5H)-one* (**3h**). Yield 59% (0.307 g); pale yellow powder; m.p. 338 °C; IR (ATR, ν (cm^−1^)): 3428 (N–H_amine_), 3289 (O–H_phenol_), 1695 (C=O_TZ_), 1331 (C–O_phenol_); ^1^H NMR (500 MHz, DMSO-*d*_6_, δ/ppm): 9.35 (s, 1H, OH), 7.88 (t, 1H, NH), 7.67 (s, 1H, –CH=), 7.35–7.71 (m, 4H, Ar–H), 6.29 (m, 1H, CH=), 5.31 (d, 2H, =CH_2_), 3.85 (m, 2H, CH_2_); ^13^C NMR (125 MHz, DMSO-*d*_6_, δ/ppm): 184.14 (C=O), 162.56 (C), 159.13 (C), 152.47 (CH), 134.75 (CH), 132.86 (C), 129.91 (2CH), 127.50 (C), 117.49 (CH_2_), 116.04 (2CH), 50.32 (CH_2_); MS (EI, 70 eV) *m*/*z* (%): 261 (M + 1, 100), 262 (M + 2, 8.7), 263 (M + 3, 3.3), 209 (1.9), 183 (1.4), 158 (1.3), 138 (5.0); Anal. Calcd. for C_13_H_12_N_2_O_2_S (260.31): C, 59.98; H, 4.65; N, 10.76; S, 12.32; Found: C, 59.79; H, 4.76; N, 10.93; S, 12.36.

*5-(3-Chlorobenzylidene)-2-(phenylamino)thiazol-4(5H)-one* (**6a**). Yield 74% (0.466 g); light brown powder; m.p. 278–280 °C; IR (ATR, ν (cm^−1^)): 3427 (N–H_amine_), 1721 (C=O_TZ_), 1157 (C–Cl); ^1^H NMR (500 MHz, DMSO-*d*_6_, δ/ppm): 11.95 (s, 1H, NH), 7.57 (s, 1H, –CH=), 7.04–7.84 (m, 9H, Ar–H); ^13^C NMR (125 MHz, DMSO-*d*_6_, δ/ppm): 187.52 (C=O), 176.43 (C), 142.15 (CH), 139.42 (C), 136.01 (C), 134.28 (C), 132.98 (C), 130.21 (CH), 129.68 (2CH), 128.77 (CH), 126.79 (CH), 126.30 (CH), 122.84 (CH), 120.71 (2CH); MS (EI, 70 eV) *m*/*z* (%): 315 (M + 1, 100), 316 (M + 2, 20.0), 317 (M + 3, 53.4), 286 (1.7), 196 (1.0), 169 (0.9), 118 (0.9); Anal. Calcd. for C_16_H_11_ClN_2_OS (314.79): C, 61.05; H, 3.52; N, 8.90; S, 10.19; Found: C, 61.09; H, 3.65; N, 8.94; S, 10.27.

*5-(2,4-Dichlorobenzylidene)-2-(phenylamino)thiazol-4(5H)-one* (**6b**). Yield 61% (0.426 g); light brown powder; m.p. 204–206 °C; IR (ATR, ν (cm^−1^)): 3434 (N–H_amine_), 1717 (C=O_TZ_), 1156 (C–Cl), 1096 (C–Cl); ^1^H NMR (500 MHz, DMSO-*d*_6_, δ/ppm): 11.97 (s, 1H, NH), 7.61 (s, 1H, –CH=), 7.06–7.80 (m, 8H, Ar–H); ^13^C NMR (125 MHz, DMSO-*d*_6_, δ/ppm): 188.02 (C=O), 177.67 (C), 140.30 (CH), 139.15 (C), 135.45 (C), 133.36 (C), 131.63 (C), 130.33 (CH), 129.52 (2CH), 128.82 (CH), 127.94 (CH), 125.66 (C), 123.74 (CH), 120.78 (2CH); MS (EI, 70 eV) *m*/*z* (%): 349 (M + 1, 100), 350 (M + 2, 13.2), 351 (M + 3, 87.6), 352 (15.8), 353 (27.0), 315 (12.0), 313 (100), 138 (0.9); Anal. Calcd. for C_16_H_10_Cl_2_N_2_OS (349.23): C, 55.03; H, 2.89; N, 8.02; S, 9.18; Found: C, 55.12; H, 2.91; N, 8.15; S, 9.34.

*5-(4-Nitrobenzylidene)-2-(phenylamino)thiazol-4(5H)-one* (**6c**). Yield 87% (0.566 g); yellow powder; m.p. 347 °C; IR (ATR, ν (cm^−1^)): 3436 (N–H_amine_), 1710 (C=O_TZ_), 1519 (NO_2_ asymmetric), 1330 (NO_2_ symmetric); ^1^H NMR (500 MHz, DMSO-*d*_6_, δ/ppm): 11.99 (s, 1H, NH), 7.69 (s, 1H, –CH=), 7.23–7.91 (m, 9H, Ar–H); ^13^C NMR (125 MHz, DMSO-*d*_6_, δ/ppm): 187.65 (C=O), 176.13 (C), 148.62 (C), 143.23 (CH), 141.85 (C), 139.99 (C), 132.44 (C), 130.09 (2CH), 129.27 (2CH), 123.95 (2CH), 122.11 (CH), 120.74 (2CH); MS (EI, 70 eV) *m*/*z* (%): 326 (M + 1, 100), 327 (M + 2, 14.2), 328 (M + 3, 6.0), 313 (8.8), 281 (100), 280 (93.0), 180 (13.4), 119 (40.9); Anal. Calcd. for C_16_H_11_N_3_O_3_S (325.34): C, 59.07; H, 3.41; N, 12.92; S, 9.86; Found: C, 59.19; H, 3.53; N, 12.98; S, 9.89.

*2-(Phenylamino)-5-((2-phenylthiazol-4-yl)methylene)thiazol-4(5H)-one* (**6d**). Yield 89% (0.647 g); light brown powder; m.p. 287–289 °C; IR (ATR, ν (cm^−1^)): 3430 (N–H_amine_), 1719 (C=O_TZ_); ^1^H NMR (500 MHz, DMSO-*d*_6_, δ/ppm): 11.92 (s, 1H, NH), 8.11 (s, 1H, C5-thiazole-H), 7.73 (s, 1H, –CH=), 7.34–8.01 (m, 10H, Ar–H); ^13^C NMR (125 MHz, DMSO-*d*_6_, δ/ppm): 187.24 (C=O), 171.76 (C), 156.61 (C), 149.98 (C), 143.85 (CH), 139.48 (C), 138.53 (C), 132.07 (C), 130.69 (2CH), 129.78 (2CH), 129.15 (2CH), 128.66 (CH), 126.31 (CH), 122.54 (CH), 120.35 (2CH); MS (EI, 70 eV) *m*/*z* (%): 364 (M + 1, 100), 365 (M + 2, 22.4), 366 (M + 3, 10.5), 338 (18.4), 261 (95.5), 243 (10.4), 246 (58.9), 219 (22.0), 218 (100), 201 (12.2); Anal. Calcd. for C_19_H_13_N_3_OS_2_ (363.46): C, 62.79; H, 3.61; N, 11.56; S, 17.64; Found: C, 62.84; H, 3.73; N, 11.67; S, 17.77.

*5-((6-Chloro-4-oxo-4H-chromen-3-yl)methylene)-2-(phenylamino)thiazol-4(5H)-one* (**6e**). Yield 94% (0.720 g); brown powder; m.p. 356 °C; IR (ATR, ν (cm^−1^)): 3429 (N–H_amine_), 1699 (C=O_TZ_), 1653 (C=O_chromone_), 1270 (C–O_chromone_), 1173 (C–Cl), 1041 (C–O_chromone_); ^1^H NMR (500 MHz, DMSO-*d*_6_, δ/ppm): 11.98 (s, 1H, NH), 7.71 (s, 1H, –CH=), 7.12–8.86 (m, 9H, Ar–H); ^13^C NMR (125 MHz, DMSO-*d*_6_, δ/ppm): 188.58 (C=O), 170.33 (C=O), 158.46 (C), 156.02 (C), 151.65 (CH), 149.39 (CH), 139.87 (C), 136.43 (C), 136.05 (CH), 130.58 (CH), 129.81 (2CH), 129.17 (C), 126.01 (C), 122.22 (CH), 120.75 (2CH), 119.30 (C), 119.12 (CH); MS (EI, 70 eV) *m*/*z* (%): 383 (M + 1, 100), 384 (M + 2, 20.2), 385 (M + 3, 31.9), 386 (M + 4, 11.3), 326 (30.03), 312 (12.7), 259 (7.8), 205 (5.9), 183 (5.5), 138 (4.8); Anal. Calcd. for C_19_H_11_ClN_2_O_3_S (382.82): C, 59.61; H, 2.90; N, 7.32; S, 8.38; Found: C, 59.66; H, 2.97; N, 7.41; S, 8.47.

*5-(3-Chlorobenzylidene)-2-(naphthalen-1-ylamino)thiazol-4(5H)-one* (**9a**). Yield 81% (0.591 g); yellow powder; m.p. 140 °C; IR (ATR, ν (cm^−1^)): 3430 (N–H_amine_), 1686 (C=O_TZ_), 1151 (C–Cl); ^1^H NMR (500 MHz, DMSO-*d*_6_, δ/ppm): 12.01 (s, 1H, NH), 7.71 (s, 1H, –CH=), 7.05–7.92 (m, 11H, Ar–H); ^13^C NMR (125 MHz, DMSO-*d*_6_, δ/ppm): 175.20 (C=O), 161.05 (C), 154.43 (CH), 144.60 (C), 135.89 (C), 134.38 (C), 134.07 (C), 132.10 (C), 130.24 (CH), 128.41 (CH), 128.11 (CH), 127.57 (CH), 126.89 (CH), 126.45 (CH), 126.26 (CH), 124.81 (CH), 123.47 (C), 121.35 (CH), 116.43 (CH), 105.63 (CH); MS (EI, 70 eV) *m*/*z* (%): 365 (M + 1, 100), 366 (M + 2, 2.5), 367 (M + 3, 19.7), 368 (M + 4, 7.7), 369 (M + 5, 1.4), 336 (3.3), 322 (2.2), 252 (4.9), 210 (2.1), 197 (64.5), 171 (10.3), 169 (100), 134 (52.2); Anal. Calcd. for C_20_H_13_ClN_2_OS (364.85): C, 65.84; H, 3.59; N, 7.68; S, 8.79; Found: C, 65.90; H, 3.66; N, 7.63; S, 8.84.

*5-(2,4-Dichlorobenzylidene)-2-(naphthalen-1-ylamino)thiazol-4(5H)-one* (**9b**). Yield 63% (0.503 g); yellow powder; m.p. 185 °C; IR (ATR, ν (cm^−1^)): 3433 (N–H_amine_), 1713 (C=O_TZ_), 1155 (C–Cl), 1099 (C–Cl); ^1^H NMR (500 MHz, DMSO-*d*_6_, δ/ppm): 12.06 (s, 1H, NH), 7.62 (s, 1H, –CH=), 7.11–8.08 (m, 10H, Ar–H); ^13^C NMR (125 MHz, DMSO-*d*_6_, δ/ppm): 175.74 (C=O), 161.42 (C), 154.35 (CH), 143.89 (C), 135.94 (C), 134.51 (C), 132.68 (C), 131.33 (C), 130.12 (CH), 128.60 (CH), 128.19 (CH), 127.76 (CH), 126.81 (CH), 126.40 (CH), 125.53 (C), 124.99 (CH), 123.93 (C), 121.17 (CH), 118.32 (CH), 105.25 (CH); MS (EI, 70 eV) *m*/*z* (%): 399 (M + 1, 90.5), 400 (M + 2, 22.2), 401 (M + 3, 100), 402 (M + 4, 17.0), 365 (31.6), 363 (100), 297 (1.0), 168 (13.2), 159 (3.4); Anal. Calcd. for C_20_H_12_Cl_2_N_2_OS (399.29): C, 60.16; H, 3.03; N, 7.02; S, 8.03; Found: C, 60.35; H, 3.08; N, 7.11; S, 8.05.

*2-(Naphthalen-1-ylamino)-5-(4-nitrobenzylidene)thiazol-4(5H)-one* (**9c**). Yield 72% (0.540 g); yellow powder; m.p. 152 °C; IR (ATR, ν (cm^−1^)): 3426 (N–H_amine_), 1706 (C=O_TZ_), 1521 (NO_2_ asymmetric), 1328 (NO_2_ symmetric); ^1^H NMR (500 MHz, DMSO-*d*_6_, δ/ppm): 12.23 (s, 1H, NH), 7.68 (s, 1H, –CH=), 7.13–8.18 (m, 11H, Ar–H); ^13^C NMR (125 MHz, DMSO-*d*_6_, δ/ppm): 174.88 (C=O), 161.31 (C), 154.10 (CH), 147.66 (C), 141.41 (C), 140.73 (C), 134.56 (C), 132.46 (C), 129.41 (2CH), 128.57 (CH), 127.32 (CH), 126.18 (CH), 125.86 (CH), 124.91 (C), 123.77 (2CH), 121.05 (CH), 118.81 (CH), 105.95 (CH); MS (EI, 70 eV) *m*/*z* (%): 376 (M + 1, 100), 378 (M + 2, 16.4), 379 (M + 3, 9.3), 330 (30.6), 316 (5.7), 208 (10.7), 180 (21.3), 170 (22.3), 169 (100), 150 (3.8), 134 (37.7); Anal. Calcd. for C_20_H_13_N_3_O_3_S (375.40): C, 63.99; H, 3.49; N, 11.19; S, 8.54; Found: C, 63.78; H, 3.52; N, 11.24; S, 8.64.

*2-(Naphthalen-1-ylamino)-5-((2-phenylthiazol-4-yl)methylene)thiazol-4(5H)-one* (**9d**). Yield 86% (0.711 g); pale yellow powder; m.p. 312 °C; IR (ATR, ν (cm^−1^)): 3429 (N–H_amine_), 1728 (C=O_TZ_); ^1^H NMR (500 MHz, DMSO-*d*_6_, δ/ppm): 12.31 (s, 1H, NH), 7.69 (s, 1H, –CH=), 7.12–8.91 (m, 13H, Ar–H + C5-thiazole-H); ^13^C NMR (125 MHz, DMSO-*d*_6_, δ/ppm): 174.67 (C=O), 169.54 (C), 161.11 (C), 149.69 (C), 142.33 (CH), 140.61 (C), 138.89 (C), 134.54 (C), 131.26 (C), 130.97 (2CH), 129.38 (2CH), 128.88 (CH), 128.79 (CH), 127.64 (CH), 126.78 (CH), 125.99 (CH), 125.07 (CH), 124.76 (C), 121.13 (CH), 118.92 (CH), 105.86 (CH); MS (EI, 70 eV) *m*/*z* (%): 414 (M + 1, 100), 415 (M + 2, 22.2), 416 (M + 3, 15.7), 387 (4.1), 261 (30.1), 246 (10.5), 218 (100), 213 (6.8), 174 (4.5), 158 (7.9), 130 (2.5); Anal. Calcd. for C_23_H_15_N_3_OS_2_ (413.51): C, 66.80; H, 3.66; N, 10.16; S, 15.51; Found: C, 66.66; H, 3.74; N, 10.19; S, 15.44.

*5-((6-Chloro-4-oxo-4H-chromen-3-yl)methylene)-2-(naphthalen-1-ylamino)thiazol-4(5H)-one* (**9e**). Yield 90% (0.779 g); orange powder; m.p. 266–268 °C; IR (ATR, ν (cm^−1^)): 3434 (N–H_amine_), 1701 (C=O_TZ_), 1649 (C=O_chromone_), 1271 (C–O_chromone_), 1170 (C–Cl), 1044 (C–O_chromone_); ^1^H NMR (500 MHz, DMSO-*d*_6_, δ/ppm): 12.51 (s, 1H, NH), 7.70 (s, 1H, –CH=), 7.11–8.74 (m, 11H, Ar–H); ^13^C NMR (125 MHz, DMSO-*d*_6_, δ/ppm): 174.21 (C=O), 160.63 (C=O), 154.38 (2C), 135.15 (CH), 134.36 (C), 131.09 (CH), 128.45 (2C), 127.49 (CH), 127.03 (C), 126.56 (CH), 126.49 (C), 125.06 (C), 124.95 (2CH), 124.49 (CH), 123.48 (2CH), 121.78 (C), 121.48 (CH), 118.76 (CH), 116.60 (CH); MS (EI, 70 eV) *m*/*z* (%): 433 (M + 1, 100), 434 (M + 2, 17.8), 435 (M + 3, 52.6), 436 (M + 4, 10.9), 413 (5.6), 401 (100), 376 (28.4), 290 (34.2), 299 (3.5), 267 (32.8), 265 (100), 237 (85.8), 205 (22.2), 165 (21.1); Anal. Calcd. for C_23_H_13_ClN_2_O_3_S (432.88): C, 63.82; H, 3.03; N, 6.47; S, 7.41; Found: C, 63.70; H, 3.01; N, 6.50; S, 7.53.

### 3.2. Antifungal Activity Assay

#### 3.2.1. Determination of Inhibition Zone Diameters

The in vitro antifungal activity was determined using the cup-plate agar diffusion method according to the Clinical and Laboratory Standards Institute (CLSI) guidelines [38]. 

Mueller-Hinton medium supplemented with 2% glucose (providing adequate growth of yeasts) and 0.5 mg/mL methylene blue (providing a better definition of the inhibition zone diameter) was used. The inoculum was prepared by suspending five representative colonies, obtained from an 18–24 h culture on non-selective nutritive agar medium, in sterile distilled water. The cell density was adjusted to the density of a 0.5 McFarland standard by spectrophotometrically measuring the absorbance at 530 nm and adding sterile distilled water as required (corresponding to a population of 1 to 5 × 10^6^ CFU/mL). A sterile swab was soaked in the suspension and then the Mueller-Hinton agar plates were inoculated by streaking the entire surface. After drying for 10–15 min, six-millimeter diameter wells were cut from the agar using a sterile cork-borer and a volume of 20 µL of each compound solution (1 mg/mL in dimethyl sulfoxide (DMSO)) were delivered into the wells (20 µg/well). Fluconazole (20 µg/well) was used as standard drug. The controls were performed with only sterile broth, overnight culture and 20 µL of DMSO. The plates were incubated at 35 °C. Zone diameters were measured to the nearest whole millimeter at a point in which there was no visible growth after 24–48 h. Results were obtained in triplicate. The solvent used for the preparation of each compound stock solution (1 mg/mL), DMSO (Merck, Darmstadt, Germany) presented no inhibitory activity against the tested fungal strains.

#### 3.2.2. Determination of MIC and MFC Values

The microorganisms used for the antimicrobial activity evaluation were obtained from the University of Agricultural Sciences and Veterinary Medicine Cluj-Napoca, Romania. The antifungal activity was evaluated against cultures of *Candida albicans* ATCC 10231, *Candida albicans* ATCC 18804, *Candida krusei* ATCC 6258, *Candida parapsilosis* ATCC 22019. The cultures were store on potato dextrose agar (Sifin, Berlin, Germany). Prior to antifungal susceptibility testing, each strain was inoculated on potato dextrose agar plates to ensure optical growth characteristics and purity. Then, yeast cells were suspended in saline and adjusted spectrophotometrically to RPMI 1640 medium. 

Stock solutions (1 mg/mL) were prepared by dissolving the test compounds and the reference antifungals (ketoconazole and fluconazole) in sterile DMSO. These solutions were stored at 4 °C. Series of double diluting solutions of the above compounds were prepared in RPMI 1640 medium obtaining final concentrations in the range of 500 to 0.015 µg/mL. 

The broth microdilution method (according to the CLSI guidelines [39]) was employed for minimum inhibitory concentration test. Media was placed into each 96 wells of the microplates. Sample solutions at high concentration (100 µg/mL) were added into the first rows of the microplates and two-fold dilutions of the compounds were made by dispensing the solutions into the remaining wells. Ten-microliter culture suspensions were inoculated into all the wells. The sealed microplates were incubated at 37 °C for 18 h. The initial density of *Candida* sp. was approximately 2 × 10^6^ colony forming units (CFU)/mL. Inoculums (density of 0.5 in McFarland scale) were prepared in a sterile solution of 0.9% NaCl. Then the tested strains were suspended in nutrient broth and RPMI 1640 media to give a final density of 2 × 10^5^ CFU/mL. Solutions of the test compounds and suspensions of fungi were inoculated onto 96 wells microplates. Growth control, sterility control and control of antifungal compounds were used. Plates were incubated under normal atmospheric conditions 25 °C for 48 h (*Candida albicans* ATCC 10231, *Candida albicans* ATCC 18804, *Candida krusei* ATCC 6258, *Candida parapsilosis* ATCC 22019), and next minimum inhibitory concentration (MIC) values have been determined by recording the optical density at 600 nm using a spectroscopically method with a microplate reader Biotek Synergy HT. The MIC was defined as the lowest concentration required arresting the growth of the fungi. For determination of minimum fungicidal concentration (MFC), a 0.01 mL aliquot of the medium drawn from the culture tubes showing no macroscopic growth at the end of the 24 h culture was subcultured on nutrient agar/potato dextrose agar plates to determine the number of vital organisms and incubated further at 37 °C for 24 h, respectively 25–30 °C for 48 h. The MFC was defined as the lowest concentration of the agent at which no colonies are observed. All MIC and MFC experiments were repeated three times.

#### 3.2.3. Statistical Analysis

The results obtained in the determination of the inhibition zone diameters assay were expressed as mean ± standard deviation (SD) of three independent experiments. Statistical comparisons between the groups were made using one-way analysis of variance (ANOVA) test. A value of *p* < 0.05 was considered to be statistically significant. Analysis was performed using standard software (Excel for Windows, version 14.0 included in Microsoft Office Professional Plus 2010).

### 3.3. Lipophilicity Assay

The lipophilic character of twenty thiazolin-4-one derivatives was assessed with the help of the principal component analysis (PCA) based on the reversed-phase thin-layer chromatography (RP-TLC) data.

#### 3.3.1. Chromatographic Procedure

The chromatographic behavior of the test compounds was studied on C_18_ silicagel bonded 60 RP-180F_254_S TLC (20 × 20 cm) plates, purchased from Merck (Darmstadt, Germany). The test compounds’ solutions (1 mg/mL) were prepared by dissolving the samples in DMSO (Merck, Darmstadt, Germany). The plates were manually spotted with 3 µL aliquots of each solution at 10 mm from the base and 5 mm from the edge of the plate, leaving a 10 mm distance between successive spots, and developed by the ascending technique, without preconditioning. Chromatography was performed in a normal developing chamber at room temperature (~24 °C), the developing distance being of 9 cm. The solvent system used as mobile phase consisted of a water-*i*-propanol mixture, with a varying content of organic modifier (*i*-propanol—concentration ranged between 45% and 65% (*v*/*v*), with 5% vol. increments). Before the development, the chamber was saturated with the mobile phase for 15 min. After being developed, the dried plates were visually inspected under the UV light (λ = 254 nm), each spot was clearly marked and its distance was manually measured in order to calculate the *R*_f_ values. The spots of the compounds **3f**, **10** and **11** were not visible in UV light, thus their *R*_f_ values could not be calculated, therefore they were excluded from this assay.

#### 3.3.2. Chromatographic Lipophilicity Parameters

Based on the *R*_f_ values, one of the most common lipophilic estimator, meaning the isocratic retention factor *R*_M_ was calculated using the Bate-Smith and Westall equations [40].

RP-TLC provides retention data in the form of *R*_f_ and the corresponding *R*_M_ values (Equation (2)) that can be used to derive chromatographic descriptors for estimating lipophilicity: *R*_M0_, *b* and PCA. The most popular lipophilicity descriptor estimated by this method is *R*_M_ and it is derived from the retention factor (*R*_f_) according to the following formula:

*R*_M_ = log[(1/*R*_f_) − 1] (2)

where *R*_f_ is calculated on the basis of migration distance of a compound and the solvent front. 

The use of RP-TLC is based on the assumed linear relationship between the molecular parameter (Equation (2)) and the standard measure of lipophilicity, logP (the logarithm of the *n*-octanol-water partition coefficient).

As *R*_M_ value (related to the molecular lipophilicity) depends linearly on the concentration of the organic modifier in the mobile phase, the value is extrapolated to pure water as mobile phase. In order to increase the accuracy of the lipophilicity determination, the *R*_M_ values extrapolated to zero organic modifier concentration (*R*_M0_) have been calculated from the linear correlation between the *R*_M_ values and the concentration of the organic component of the mobile phase:

*R*_M_ = *R*_M0_ + bc (3)

where *R*_M0_ is the lipophilicity estimation parameter, *R*_M_ is the retention of the solute, b is the slope, and c is the concentration of the mobile phase organic modifier (*i*-propanol).

The intercept (*R*_M0_) in Equation (3) represents the extrapolated *R*_M_ values at 0% organic modifier. In other words, the intercept determined using this equation can be considered an estimation of the partitioning of compounds between the nonpolar stationary phase and the aqueous system, hence all compounds studied can be compared on the basis of their lipophilicity determined this way [41].

The lipophilic properties of the investigated compounds were evaluated by using the principal component analysis (PCA, XL-STAT). For a better interpretation of the obtained results, these were correlated with cLogP values of the tested compounds, which were generated by the ChemBioDraw 11.0 software.

#### 3.3.3. Principal Component Analysis (PCA)

PCA, also known as eigenvector analysis, is a statistical procedure used to represent in an economic way the location of the objects in a reduced coordinate system where instead of *n*-axes (corresponding to *n* variables) only *p* (*p* ≤ *n*) can usually be used to describe the data set with maximum possible information. The new variables are called principal components and they are given by the linear combination of the *n* real variables [42].

PCA was performed on the obtained retention parameters with the help of XL-STAT extension. It displayed objects (the tested compounds) in a reduced space by finding a direction (first principal component) that best preserved the scatter of the observations (100 × *R*_f_ values) in the multidimensional space. PCA gave the coordinates (scores) of the studied compounds and also the loadings of the variables (solvents) on the principal components.

### 3.4. Virtual Screening

Identification of good “hits” (lead/drug-like molecules) is the first critical step in the drug discovery and development process. The qualities of the “hits” may dramatically set the stage for subsequent efforts to improve their therapeutic efficacy through potency against their designated target, their selectivity against related targets (including here protein binding specificity versus promiscuity), adequate pharmacokinetics, and lowering toxicity and their side effects [43].

The synthesized thiazolin-4-one derivatives and the reference compounds (fluconazole and ketoconazole) were screened in silico for both prediction of ADME-Tox properties (including here lipophilicity) and the binding modes with their designated targets. An academic license of MarvinSketch was used for drawing, displaying of 2D structures and 3D optimization of all screened compounds and generations of input files (SDF and Tripos MOL2 file formats) for virtual screening, MarvinSketch 15.1.5.0, 2015, ChemAxon (https://www.chemaxon.com/). 

#### 3.4.1. ADME-Tox Predictions

Achieving the desired specificity and activity alone is not a self-sufficient goal to produce high-quality leads or drug candidates. Therefore, pharmacokinetic and metabolic characteristics should be taken into consideration as early as possible hence the major reasons preventing many candidates from reaching market are the side effects, respectively the inappropriate ADME-Tox properties. Therefore, from an economical point-of-view, it is desirable that compounds with inappropriate ADME-Tox properties are optimized or removed from the early discovery phase rather than during the expensive drug development phases.

The use of cheminformatics to predict the ADME-Tox properties can provide helpful guidance on absorption, plasma clearance and tissue distribution, activity at the level of central nervous system (penetration of compounds through the BBB), various metabolic effects and toxicity aspects. In this respect, FAF-Drugs3 [30], a web-based software, hosted on the public domain of ***The Ressource Parisienne en Bioinformatique Structurale (RPBS)*** (http://fafdrugs3.mti.univ-paris-diderot.fr/), was used to screen all thiazolin-4-one derivatives and the reference compounds for ADME-Tox properties.

Prior to screening, the input SDF files were formatted according to the software’s requirements using Bank Formatter (the files formatter service of FAF-Drugs3) and XLOGP3 was chosen as method to estimate lipophilicity (and the derived ADME-Tox descriptors) due to its prediction accuracy. VS was carried out using a series of FAF-Drugs3’s built-in filters for lead-likeness, drug-likeness, activity at CNS level [33], detection of non-peptidic inhibitors of protein–protein interactions, detection of undesirable moieties and substructures involved in toxicity problems [35,36], covalent inhibitors, PAINS and customized filters for safety profiling [35]. 

The chosen *Lead-Like Soft filter* uses well-known lead-like descriptors, while the *Drug-Like Soft filter* is based on physico-chemical, molecular properties and bioavailability rules, widely used for drug discovery—both *soft filters* use a built-in statistical analysis of drugs [30] extracted from the ***e-Drugs3D library*** for the threshold values of descriptors. 

PPIs are essential to a healthy life, but aberrant PPIs contribute to many diseases and therefore represent a very populated class of essentially untouched targets for rational drug design. In this respect, FAF-Drugs3 uses a decision tree constructed with the help of two trained Dragon descriptors (***Ui*** and ***RDF070m***). 

For the detection of the undesirable moieties and substructures involved in toxicity problems, the FAF-Drugs3 built-in filters based on a comprehensive literature data were used. As a particular threshold of the software, with direct interest for our work, is the acceptance of up to 3 nitro groups, otherwise well-known structural alerts, because several approved and experimental drugs (e.g., nitroimidazole antibiotics) have at least one nitro group and because this group can be replaced during the optimization process. Similar to the detection of UMSs, FAF-Drugs3 uses built-in filters to identify covalent inhibitors.

The detection of the possible frequent hitters, compounds that appear in many biochemical high throughput screens—PAINS moieties, was done using three filters (A, B and C), in order to detect problematic compound classes in the ***WEHI* (The Walter and Eliza Hall Institute of Medical Research, Parkville, Australia) *93K HTS library*** that contain over 150 analogs (filter A), from 15 to 149 analogs (filter B), respectively, 1 to 14 analogs (filter C) [30]. 

A series of customized filters developed by pharmaceutical companies were used to assess the safety profiling: the GSK 4/400 rule, the Pfizer 3/75 rule, estimation of phospholipidosis induction (directly linked with the molecular substructure of compounds) and the MedChem rules rating [35]. The MedChem rules is a set of 275 rules developed by Eli Lilly and Company (Indianapolis, IN, USA), to identify compounds that may interfere with biological assays, allowing their removal from screening sets. For this particular VS run, the MedChem rules were used with the regular settings (100-demerit cutoff). 

#### 3.4.2. Molecular Docking Studies

The thiazolin-4-one derivatives and the corresponding reference compounds (generated as Tripos MOL2 files) were docked in silico against their corresponding target (the same species used for determination of MIC and MFC values were considered): a fungal enzyme (lanosterol 14-α demethylase). Due to the lack of experimentally determined high-resolution crystal structures from ***Research Collaboratory for Structural Bioinformatics—Protein Data Bank*** (RCSB-PDB: http://www.rcsb.org/), as best recommended for docking [44], a homology model was used for the target protein. 

The homology model for lanosterol-14α-demethylase was generated with ***SWISS-MODEL*** (a fully automated protein structure homology-modeling server, accessible via ExPASy web server: http://www.expasy.org/) [45] starting from the corresponding amino acid sequence (Accession Code: P10613) of the enzyme for *Candida albicans* (strain SC5314/ATCC MYA-2876) obtained from the ***Universal Protein Resource (UniProt)*** (http://www.uniprot.org/) and using as template a validated experimental structure from *Saccharomyces cerevisiae* (PDB ID: 4WMZ) [45].

Lanosterol 14-α demethylase (cytochrome P450 14α-lanosterol-demethylase) catalyzes the C14-demethylation of lanosterol which is critical for ergosterol biosynthesis (transforms lanosterol into 4,4′-dimethyl cholesta-8,14,24-triene-3-beta-ol). Therefore, lanosterol 14-α demethylase is an enzyme with a crucial role in sterol biosynthesis in eukaryotes, being thus a druggable protein for antifungal design and development of new inhibitors. The active site of enzyme includes a triazole ring perpendicularly positioned to the porphyrin plane with a ring nitrogen atom coordinated to the Hem iron. Docking of thiazolin-4-one derivatives and reference compounds (fluconazole and ketoconazole) against the generated homology model for lanosterol-14α-demethylase was carried out setting an extended search space (X:26.610; Y:18.853; Z:19.715), with a volume bigger than 27.000 Å^3^ and manually increasing the exhaustiveness to 80 in order to improve docking accuracy. 

## 4. Conclusions

Twenty-three thiazoline-4-ones were synthesized, of which 18 are new 5-arylidene-thiazoline-4-one derivatives. The newly synthesized compounds were characterized by quantitative elemental analysis, IR, ^1^H NMR, ^13^C NMR and MS. All compounds were evaluated for their antifungal activity against four strains of *Candida* and the compounds’ lipophilicity was assessed. Most of the compounds presented excellent anti-*Candida* properties, two of them (**10** and **9b**) being 250-fold more active than ketoconazole and over 500-fold more active than fluconazole. For all the studied compounds, it was observed a lipophilicity augmentation with the increase of the molar mass of the substituent from position 2 of the thiazolin-4-one core, and also, by introducing an arylidene or hetarylidene rest in position 5. A comprehensive VS was carried out for ADME-Tox profiling of the thiazolin-4-one derivatives and it can be concluded that based on the predicted values of physico-chemical descriptors, almost all derivatives are drug-like small-molecules (except **9b**), non-inducer of phospholipidosis and with good predictions for oral bioavailability according to RO5, Veber’s rule and Egan’s rule, thus making them suitable for systemic use. Most of the drug-like derivatives forecasted good activity at CNS level (except **3c**, **6c**, **9b**, **9c** and **10**), but all require further structural optimization due multiple safety alerts. Therefore, compound **11** appears to be the safest drug-like derivative since it has only two alerts (PPI unfriendly and a warning from Pfizer 3/75 rule).

On the other hand, some of thiazolin-4-one derivatives were also qualified as potential leads (**2**, **3a**, **3c**–**h**, **5**, **6c**, **8** and **11**) able to penetrate BBB (except **3c** and **6c**) and with good predictions for oral bioavailability. However, all those lead-like compounds require further optimization in the drug development process. In regards to this aspect, we can identify as “hits” compounds **5** and **8** (both free of UMSs, covalent inhibitors and PAINS—according to all three PAINS’s filters, but PPI unfriendly and with different safety concerns according to the MedChem rules); compound **6c** (the only lead compound which is PPI friendly), and compound **11** (with the already discussed safety concerns as drug candidate). 

Regarding the thiazolin-4-one derivatives inhibitory activity as antifungals we can summarize that from the identified “hits”, two derivatives (**5** and **11**) are weaker inhibitors than both reference drugs (**Flu** and **Ket**), meanwhile **6c** and **8** have a better binding affinity than **Flu**, but with lesser safety concerns than **Ket**—both virtually predicted and referred in the literature [46]. Moreover, three of the identified “hits” (**5**, **8** and **11**) block the same entry of the active site of lanosterol 14α-demethylase, while only **6c** binds deeply inserted in the active site.

## Figures and Tables

**Table 1:** The antifungal activity of the synthesized thiazolin-4-one derivatives (inhibition zone diameters (mm)).

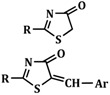		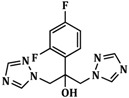	
2, 5, 8, 10, 11	Fluconazole	3a–h, 6a–e, 9a–e	
Compound	R	Ar	*C. albicans* ATCC 10231
**2**	CH_2_=CH–CH_2_–NH–	-	20 ± 0.2
**5**	C_6_H_5_–NH–	-	**22 ± 0.5**
**8**	α-C_10_H_7_–NH–	-	**24 ± 1**
**10**	CH_3_–NH–	-	**22 ± 1**
**11**	C_6_H_5_–	-	18 ± 1
**3a**	CH_2_=CH–CH_2_–NH–	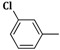	20 ± 0.5
**6a**	C_6_H_5_–NH–		20 ± 0.5
**9a**	α-C_10_H_7_–NH–		18 ± 1
**3b**	CH_2_=CH–CH_2_–NH–	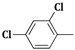	20 ± 0.5
**6b**	C_6_H_5_–NH–		20 ± 1
**9b**	α-C_10_H_7_–NH–		18 ± 0.2
**3c**	CH_2_=CH–CH_2_–NH–	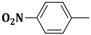	18 ± 1
**6c**	C_6_H_5_–NH–		20 ± 1
**9c**	α-C_10_H_7_–NH–		20 ± 0.5
**3d**	CH_2_=CH–CH_2_–NH–	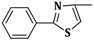	20 ± 0.2
**6d**	C_6_H_5_–NH–		20 ± 1
**9d**	α-C_10_H_7_–NH–		20 ± 1
**3e**	CH_2_=CH–CH_2_–NH–	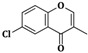	20 ± 0.5
**6e**	C_6_H_5_–NH–		16 ± 1
**9e**	α-C_10_H_7_–NH–		**22 ± 0.5**
**3f**	CH_2_=CH–CH_2_–NH–	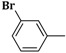	**22 ± 0.2**
**3g**	CH_2_=CH–CH_2_–NH–	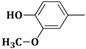	**22 ± 0.2**
**3h**	CH_2_=CH–CH_2_–NH–	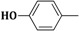	20 ± 0.5
**Fluconazole**			**22 ± 0.5**

The value obtained for each compound represents the mean of three independent measurements ± SD. The values obtained for the most active compounds are marked in bold.

**Table 2:** Minimum Inhibitory Concentration (MIC) (µg/mL) and Minimum Fungicidal Concentration (MFC) (µg/mL) of the thiazolin-4-one derivatives.

Samples	*C. albicans* ATCC 10231		*C. albicans* ATCC 18804		*C. krusei* ATCC 6258		*C. parapsilosis* ATCC 22019	
	MIC	MFC	MIC	MFC	MIC	MFC	MIC	MFC
**2**	**0.12**	**0.24**	**0.24**	**0.48**	**0.24**	**0.48**	**0.12**	**0.24**
**5**	**0.24**	**0.48**	**0.12**	**0.24**	**0.06**	**0.12**	**0.06**	**0.12**
**8**	**0.12**	**0.24**	**0.12**	**0.24**	**0.06**	**0.12**	**0.03**	**0.12**
**10**	**0.015**	**0.03**	**0.015**	**0.03**	**0.015**	**0.015**	**0.015**	**0.015**
**11**	**0.24**	**0.48**	**0.12**	**0.24**	**0.12**	**0.48**	**0.06**	**0.12**
**3a**	31.25	**31.25**	**15.62**	**31.25**	**15.62**	**31.25**	**7.81**	**15.62**
**3b**	31.25	**31.25**	**15.62**	**15.62**	**15.62**	**31.25**	**7.81**	**15.62**
**3c**	31.25	**31.25**	31.25	**31.25**	**15.62**	**15.62**	15.62	31.25
**3d**	31.25	62.5	31.25	62.5	**15.62**	**31.25**	**7.81**	**15.62**
**3e**	31.25	62.5	**15.62**	**31.25**	**15.62**	**31.25**	**7.81**	**15.62**
**3f**	1.95	**3.9**	**7.81**	**15.62**	**3.9**	**7.81**	**1.95**	**3.9**
**3g**	0.48	**1.95**	**0.48**	**0.97**	**0.48**	**1.95**	**0.24**	**0.48**
**3h**	0.24	**0.48**	**0.48**	**0.97**	**0.24**	**0.97**	**0.12**	**0.97**
**6a**	15.62	**31.25**	**7.81**	**15.62**	**7.81**	**3.9**	**3.9**	**3.9**
**6b**	15.62	**31.25**	**15.62**	**31.25**	**7.81**	**7.81**	**3.9**	**3.9**
**6c**	0.97	**1.95**	**1.95**	**3.9**	**0.97**	**1.95**	**0.97**	**1.95**
**6d**	3.9	**7.81**	**7.81**	**15.62**	**7.81**	**15.62**	**3.9**	**7.81**
**6e**	**1.95**	**3.9**	**0.97**	**3.9**	**0.97**	**3.9**	**0.48**	**0.97**
**9a**	**3.9**	**7.81**	**1.95**	**7.81**	**0.48**	**0.97**	**0.48**	**0.97**
**9b**	**0.015**	**0.03**	**0.015**	**0.03**	**0.015**	**0.015**	**0.015**	**0.015**
**9c**	**1.95**	**3.9**	**1.95**	**3.9**	**0.97**	**1.95**	**0.03**	**0.06**
**9d**	**7.81**	**15.62**	**3.9**	**3.9**	**0.24**	**0.48**	**0.12**	**0.24**
**9e**	**7.81**	**15.62**	**1.95**	**3.9**	**1.95**	**3.9**	**1.95**	**3.9**
**Fluconazole**	**15.62**	**31.25**	**15.62**	**31.25**	**15.62**	**31.25**	**7.81**	**15.62**
**Ketoconazole**	**7.81**	**15.62**	**7.81**	**15.62**	**7.81**	**15.62**	**3.9**	**7.81**
**Inoculum control**	+++	-	+++	-	+++	-	+++	-
**Broth control**	No growth	-	No growth	-	No growth	-	No growth	-

- indicates the compound has no inhibitory activity; +++ indicates growth in all concentrations. The values obtained for the most active compounds are marked in bold.

**Table 3:** The eigenvalues of the five components (*i*-propanol concentration).

Component	Eigenvalue	Difference	Variability (%)	Cumulative (%)
**1**	4.830	4.671	96.609	96.609
**2**	0.159	0.153	3.175	99.784
**3**	0.006	0.002	0.123	99.907
**4**	0.004	0.003	0.071	99.978
**5**	0.001	-	0.022	100.000

**Table 4:** Eigenvectors associated with the first two principal components.

Component	Eigenvector	
	*P*_1_	*P*_2_
**1**	0.437	0.693
**2**	0.450	0.361
**3**	0.452	−0.240
**4**	0.451	−0.310
**5**	0.446	−0.485

**Table 5:** Regression data, scores of the two first principal components and cLogP values for the studied compounds.

Compound	*R*_M0_	b	*R*^2^	*P*_1_	*P*_2_	cLogP
**2**	0.0414	−1.1059	0.9014	4.335	0.879	0.779
**3a**	2.0801	−3.6152	0.9413	−0.090	−0.268	3.725
**3b**	2.2398	−3.6102	0.9716	−1.263	−0.062	4.438
**3c**	1.7553	−3.314	0.9537	1.049	−0.259	2.755
**3d**	2.2119	−3.7366	0.9427	−0.549	−0.241	3.454
**3e**	2.2869	−3.9184	0.9455	−0.349	−0.354	2.965
**3g**	0.6734	−1.9546	0.9598	3.365	0.453	2.1942
**3h**	1.1465	−2.6897	0.9407	2.905	0.056	2.345
**5**	0.9459	−2.2931	0.9239	2.796	0.238	0.851
**6a**	2.1738	−3.9258	0.9316	0.485	−0.534	4.86
**6b**	2.4679	−4.0118	0.9544	−1.253	−0.186	5.573
**6c**	1.7388	−3.3696	0.9487	1.394	−0.304	3.89
**6d**	2.1996	−3.8092	0.9346	−0.168	−0.359	4.589
**6e**	2.22	−3.9066	0.9518	0.079	−0.443	4.1
**8**	1.6225	−3.0931	0.9344	1.125	−0.163	2.025
**9a**	2.5309	−4.1292	0.9555	−1.227	−0.239	6.034
**9b**	2.9283	−4.3675	0.9661	−2.876	0.232	6.747
**9c**	2.7789	−4.1555	0.9654	−2.722	0.228	5.264
**9d**	3.287	−4.6935	0.8744	−3.746	0.618	5.763
**9e**	2.3983	−3.319	0.9422	−3.292	0.708	5.274

**Table 6:** The results of VS carried out for lead-like and drug-like descriptors.

ID	MW (Da)	LogP	H_BA_	H_BD_	tPSA (Å^2^)	RtB	RiB	R_s_	M_x_S	C_s_	HA	H/C	Crg	T_Crg_	SC
**2**	156.21	0.79	3	1	66.76	3	7	1	5	6	4	0.67	1	−1	0
**3a**	278.76	3.54	3	1	66.76	4	14	2	6	13	5	0.38	0	0	0
**3b**	313.20	4.17	3	1	66.76	4	14	2	6	13	6	0.46	0	0	0
**3c**	289.31	2.74	6	1	112.58	5	16	2	6	13	7	0.54	0	0	0
**3d**	327.42	3.64	4	1	107.89	5	19	3	6	16	6	0.38	0	0	0
**3e**	346.79	3.18	5	1	96.97	4	20	2	10	16	7	0.44	0	0	0
**3f**	323.21	3.61	3	1	66.76	4	14	2	6	13	5	0.38	0	0	0
**3g**	290.34	2.53	5	2	96.22	5	14	2	6	14	6	0.43	0	0	0
**3h**	260.31	2.56	4	2	86.99	4	14	2	6	13	5	0.38	0	0	0
**5**	192.24	1.70	3	1	73.08	2	12	2	6	9	4	0.44	1	-1	0
**6a**	314.79	4.46	3	1	66.76	3	19	3	6	16	5	0.31	0	0	0
**6b**	349.23	5.09	3	1	66.76	3	19	3	6	16	6	0.38	0	0	0
**6c**	325.34	3.66	6	1	112.58	4	21	3	6	16	7	0.44	0	0	0
**6d**	363.46	4.56	4	1	107.89	4	24	4	6	19	6	0.32	0	0	0
**6e**	382.82	4.09	5	1	96.97	3	25	3	10	19	7	0.37	0	0	0
**8**	242.30	2.95	3	1	73.08	2	17	2	10	13	4	0.31	1	−1	0
**9a**	364.85	5.71	3	1	66.76	3	24	3	10	20	5	0.25	0	0	0
**9b**	399.29	*6.34* *	3	1	66.76	3	24	3	10	20	6	0.30	0	0	0
**9c**	375.40	4.91	6	1	112.58	4	26	3	10	20	7	0.35	0	0	0
**9d**	413.51	5.81	4	1	107.89	4	29	4	10	23	6	0.26	0	0	0
**9e**	432.88	5.34	5	1	96.97	3	30	3	10	23	7	0.30	0	0	0
**10**	130.17	0.15	3	1	66.76	1	6	1	5	4	4	1.00	1	−1	0
**11**	177.22	1.98	2	0	54.73	1	12	2	6	9	3	0.33	0	0	0
**Flu**	306.27	0.52	7	1	81.65	5	16	3	6	13	9	0.69	0	0	1
**Ket**	531.43	3.26	8	0	69.06	7	30	5	6	26	10	0.38	0	0	2
**DLS_tv_**	100–600	−3–6	≤12	≤5	≤180	≤11	≤30	≤6	≤18	3–35	1–15	0.1–1.1	≤3	−2–2	-
**LLS_tv_**	150–400	−3–4	≤7	≤4	≤160	≤9	≤30	≤4	≤18	3–35	1–15	0.1–1.1	≤3	−2–2	≤2
**CNS_tv_**	135–582	−0.2–6.1	≤5	≤3	3–118	-	-	-	-	-	-	-	-	-	-

**DLS_tv_**: Threshold values of the *Drug-Like Soft* filter; **LLS_tv_**: Threshold values of the *Lead-Like Soft* filter; **CNS_tv_**: Threshold values of the *CNS* filter; Underlined values: Overpass the thresholds for lead-likeness filters; * bold, italic values: Overpass the thresholds for drug-likeness filters; Red values: Overpass the thresholds for CNS activity filters (penetration of BBB).

**Table 7:** Binding affinity of the tested compounds (best poses) towards lanosterol-14α-demethylase.

ID	BA (kcal/mol)	H-Bonds: Best Pose Interaction(s) with Lanosterol-14α-demethylase	
		AA Residue(s) of Target	Interacting Atom(s) of the Ligand (#atom)
**2**	−5.4	Asn440	Oxygen (#6)
**3a**	**−8.1**	Asn440	Nitrogen (#2)
**3b**	**−7.9**	N/A	N/A(N/A)
**3c**	**−7.9**	Asn440	Nitrogen (#2)
**3d**	**−8.4**	N/A	N/A(N/A)
**3e**	**−8.4**	Met508	Nitrogen (#2)
**3f**	**−8.0**	Asn440	Nitrogen (#2)
**3g**	**−7.6**	Asn440	Nitrogen (#2)
**3h**	**−7.6**	Asn440	Nitrogen (#2)
**5**	−7.4	Tyr460 Ser436	Hydrogen (#16) Oxygen (#6)
**6a**	**−9.5**	Ile304	Hydrogen (#22)
**6b**	**−8.8**	N/A	N/A(N/A)
**6c**	**−8.8**	N/A	N/A(N/A)
**6d**	**−10.1**	N/A	N/A(N/A)
**6e**	**−10.3**	N/A	N/A(N/A)
**8**	**−9.4**	Asn440	Oxygen (#6)
**9a**	**−10.0**	N/A	N/A(N/A)
**9b**	**−10.3**	His468	Nitrogen (#2)
**9c**	**−10.2**	N/A	N/A(N/A)
**9d**	**−11.2**	N/A	N/A(N/A)
**9e**	**−11.4**	N/A	N/A(N/A)
**10**	−4.6	Asn440	Oxygen (#6)
**11**	−6.9	N/A	N/A(N/A)
**Flu**	−7.6	Thr311	Nitrogen (#8)
**Ket**	−10.0	N/A	N/A(N/A)

N/A: Not available/assigned; #atom: Atoms numbering; **Bold values**: The best binding affinities (similar to or higher than those of the reference compounds).

## Supplementary Materials

Supplementary materials can be found at www.mdpi.com/1422-0067/18/1/177/s1.
