# Supplementary material for: Design, Synthesis and Antifungal Activity Evaluation of New Thiazolin-4-ones as Potential Lanosterol 14α-Demethylase Inhibitors
Source: Int J Mol Sci. 2017 Jan 17;18(1):177. doi: 10.3390/ijms18010177 (PMC5297809; doi:10.3390/ijms18010177)
Supplement: Supplementary file 1 [file ijms-18-00177-s001.pdf]

Supplementary Materials: Design, Synthesis and Antifungal Activity Evaluation of New Thiazolin-4-ones as Potential Lanosterol 14α-Demethylase Inhibitors

Anca Stana, Dan C. Vodnar, Radu Tamaian, Adrian Pîrnău, Laurian Vlase, Ioana Ionuț, Ovidiu Oniga and Brîndușa Tiperciuc

Table S1. The results of VS carried out for detection of non-peptidic inhibitors of PPI and UMSs (with prediction of toxicity risks).

ID	PPI Friendly	UMSs		
		**Detected Functional Group**	**Main Mechanism**	**Alternative Mechanisms**
**2**	No	LR: Michael acceptors (double bonds)	Warhead	-
		LR: terminal vinyl	Problematic	-
**3a**	No	HR: Michael acceptors (double bonds)	Warhead	-
		LR: terminal vinyl	Problematic	-
**3b**	No	HR: Michael acceptors (double bonds)	Warhead	-
		LR: terminal vinyl	Problematic	-
**3c**	No	HR: Michael acceptors (double bonds)	Warhead	-
		LR: nitro	Reactive	Binder
		LR: nitrobenzene	Reactive	CovB, DNAB
		LR: terminal vinyl	Problematic	-
**3d**	No	HR: Michael acceptors (double bonds)	Warhead	-
		LR: terminal vinyl	Problematic	-
		LR: thiazole	Oxidative	-
**3e**	No	HR: Michael acceptors (double bonds)	Warhead	-
		LR: terminal vinyl	Problematic	-
**3f**	No	HR: Michael acceptors (double bonds)	Warhead	-
		LR: terminal vinyl	Problematic	-
**3g**	No	HR: Michael acceptors (double bonds)	Warhead	-
		LR: phenol		
		LR: terminal vinyl	Problematic	-
**3h**	No	HR: Michael acceptors (double bonds)	Warhead	-
		LR: phenol		
		LR: terminal vinyl	Problematic	-
**5**	No	not detected	-	-
**6a**	No	LR: Michael acceptors (double bonds)	Warhead	-
**6b**	No	LR: Michael acceptors (double bonds)	Warhead	-
**6c**	Yes	LR: Michael acceptors (double bonds)	Warhead	-
		LR: nitro	Reactive	Binder
		LR: nitrobenzene	Reactive	Cov_B, DNA_B
**6d**	Yes	LR: Michael acceptors (double bonds)	Warhead	-
		LR: thiazole	Oxidative	-
**6e**	Yes	LR: Michael acceptors (double bonds)	Warhead	-
**8**	No	not detected	-	-
**9a**	Yes	LR: Michael acceptors (double bonds)	Warhead	-
**9b**	Yes	LR: Michael acceptors (double bonds)	Warhead	-
**9c**	Yes	LR: Michael acceptors (double bonds)	Warhead	-
		LR: nitro	Reactive	Binder
		LR: nitrobenzene	Reactive	Cov_B, DNA_B
**9d**	Yes	LR: Michael acceptors (double bonds)	Warhead	-
		LR: thiazole	Oxidative	-
**9e**	Yes	LR: Michael acceptors (double bonds)	Warhead	-

Table S1. *Cont.*

ID	PPI Friendly	UMSs		
		Detected Functional Group	Main Mechanism	Alternative Mechanisms
**10**	No	-	-	-
**11**	No	-	-	-
**Flu**	No	HR: triazole	$CYP450_I$	$CYP450_B$
**Ket**	Yes	LR: cyclic acetal<br>LR: imidazole	Problematic<br>$CYP\text{-}OX_S$	-<br>$CYP450_B$

LR: low risk UMSs detected; HR: high risk UMSs detected; - Indicates not detected UMSs/mechanisms; Warhead: functional group responsible for electrophilic protein-reactive false positives; Problematic: functional group with inherent or indirect toxicity; Reactive (metabolite): structural alert requiring metabolism to generate a reactive metabolite (can form adducts with endogenous biomolecules); Binder: indicate the existence of all following binding mechanisms: covalent binding (Cov_B: problematic group involved in covalent binding with biological macromolecules), CYP450 binding ($CYP45_B$: structural alerts exhibiting tight binding to CYP450s enzymes), DNA binding (DNA_B: structural alert with a propensity for DNA binding); Oxidative: oxidative ring scission catalyzed by P450 enzymes resulting in the formation of the corresponding α; $CYP450_I$: structural alert requiring metabolism to generate a reactive metabolite which inhibits P450 enzymes; $CYP\text{-}OX_S$: structural alert referring at a reactive metabolite used as substrate for CYP oxidation

Table S2. The results of VS carried out for detection of covalent inhibitors and PAINS (with resolution from the build-in decisional three).

ID	Detected Covalent Inhibitors	PAINS		
		Filter A	Filter B	Filter C
**2**	Michael acceptors (double bonds)	Accepted	Accepted	Accepted
**3a**	Michael acceptors (double bonds)<br>α, β-unsaturated carbonyl	Accepted	Intermediate (90)	Accepted
**3b**	Michael acceptors (double bonds)<br>α, β-unsaturated carbonyl	Accepted	Intermediate (90)	Accepted
**3c**	Michael acceptors (double bonds)<br>α, β-unsaturated carbonyl	Accepted	Intermediate(90)	Accepted
**3d**	Michael acceptors (double bonds)<br>α, β-unsaturated carbonyl	Accepted	Intermediate (90)	Accepted
**3e**	Michael acceptors (double bonds)<br>α, β-unsaturated carbonyl	Accepted	Intermediate (90)	Accepted
**3f**	Michael acceptors (double bonds)<br>α, β-unsaturated carbonyl	Accepted	Intermediate (90)	Accepted
**3g**	Michael acceptors (double bonds)<br>α, β-unsaturated carbonyl	Accepted	Intermediate (90)	Accepted
**3h**	Michael acceptors (double bonds)<br>α, β-unsaturated carbonyl	Accepted	Intermediate (90)	Accepted
**5**	-	Accepted	Accepted	Accepted
**6a**	Michael acceptors (double bonds)<br>α, β-unsaturated carbonyl	Accepted	Intermediate (90)	Accepted
**6b**	Michael acceptors (double bonds)<br>α, β-unsaturated carbonyl	Accepted	Intermediate (90)	Accepted
**6c**	Michael acceptors (double bonds)<br>α, β-unsaturated carbonyl	Accepted	Intermediate (90)	Accepted
**6d**	Michael acceptors (double bonds)<br>α, β-unsaturated carbonyl	Accepted	Intermediate (90)	Accepted
**6e**	Michael acceptors (double bonds)<br>α, β-unsaturated carbonyl	Accepted	Intermediate (90)	Accepted
**8**	-	Accepted	Accepted	Accepted

Table S2. *Cont.*

ID	Detected Covalent Inhibitors	PAINS		
		**Filter A**	**Filter B**	**Filter C**
**9a**	Michael acceptors (double bonds) α, β-unsaturated carbonyl	Accepted	Intermediate [(90)]	Accepted
**9b**	Michael acceptors (double bonds) α, β-unsaturated carbonyl	Accepted	Intermediate [(90)]	Accepted
**9c**	Michael acceptors (double bonds) α, β-unsaturated carbonyl	Accepted	Intermediate [(90)]	Accepted
**9d**	Michael acceptors (double bonds) α, β-unsaturated carbonyl	Accepted	Intermediate [(90)]	Accepted
**9e**	Michael acceptors (double bonds) α, β-unsaturated carbonyl	Accepted	Intermediate [(90)]	Accepted
**10**	-	Accepted	Accepted	Accepted
**11**	-	Accepted	Accepted	Accepted
**Flu**	-	Accepted	Accepted	Accepted
**Ket**	-	Intermediate [(246)]	Accepted	Accepted

- Indicates not detected covalent inhibitors; Accepted: compounds with no structural alerts for PAINS and, concomitantly, satisfying the physicochemical filter (those results are displayed in Table 6); Intermediate: compounds which embeds low-risk structural PAINS alerts with a number of occurrences below the threshold (to not be confused with the intermediate thiazolin-4-one derivatives, which are reaction intermediates); [(90)] PAIN substructure filter name: ene_five_het_B; [(246)] PAIN substructure filter name: anil_di_alk_C.

Table S3. The results of VS carried out for drug safety profiling.

ID	4/400 Rule	3/75 Rule	Phospholipidosis	MedChem Rules (Rule Name)
**2**	good	warning	non inducer	isothiourea_sulfonamide
**3a**	good	bad	non inducer	isothiourea_sulfonamide
**3b**	good	bad	non inducer	isothiourea_sulfonamide
**3c**	good	good	non inducer	isothiourea_sulfonamide
**3d**	good	warning	non inducer	isothiourea_sulfonamide
**3e**	good	warning	non inducer	isothiourea_sulfonamide
**3f**	good	bad	non inducer	isothiourea_sulfonamide
**3g**	good	good	non inducer	isothiourea_sulfonamide
**3h**	good	good	non inducer	isothiourea_sulfonamide
**5**	good	warning	non inducer	isothiourea_sulfonamide
**6a**	good	bad	non inducer	isothiourea_sulfonamide
**6b**	good	bad	non inducer	isothiourea_sulfonamide
**6c**	good	warning	non inducer	isothiourea_sulfonamide
**6d**	good	warning	non inducer	isothiourea_sulfonamide
**6e**	good	warning	non inducer	isothiourea_sulfonamide
**8**	good	warning	non inducer	amino_naphthalene
**9a**	good	bad	non inducer	amino_naphthalene
**9b**	good	bad	non inducer	amino_naphthalene
**9c**	good	warning	non inducer	amino_naphthalene
**9d**	bad	warning	non inducer	amino_naphthalene
**9e**	bad	warning	non inducer	amino_naphthalene
**10**	good	warning	non inducer	isothiourea_sulfonamide
**11**	good	warning	non inducer	-
**Flu**	good	good	non inducer	-
**Ket**	good	bad	inducer	too_many_atoms<br>aniline_no_h_newd<br>acetal_both_in_ring

- Indicates not detected substructures according MedChem rules; isothiourea_sulfonamide: this rule is an acylating-class rule and is referring to the presence of isothiourea sulfonylated on imine nitrogen; amino_naphthalene: this rule is a nuisance-class rule and is referring at a type of interference that is not amenable to the substructure search, developed to flag interfering compounds that passed the substructure rules; too_many_atoms: this rule is a miscellaneous-class rule and is referring to the presence of over 25 non-hydrogen atoms; positive: this rule is an miscellaneous-class rule and is referring to the 50 demerits for each positive charge >1; aniline_no_h_newd: this rule is a nitrogen-class rule and is referring to the presence of aniline (cannot have *ortho* or *para* electron withdrawing group); acetal_both_in_ring: this rule is an aldehyde-class rule and is referring to the presence of acetal with both oxygen or sulphur in ring.

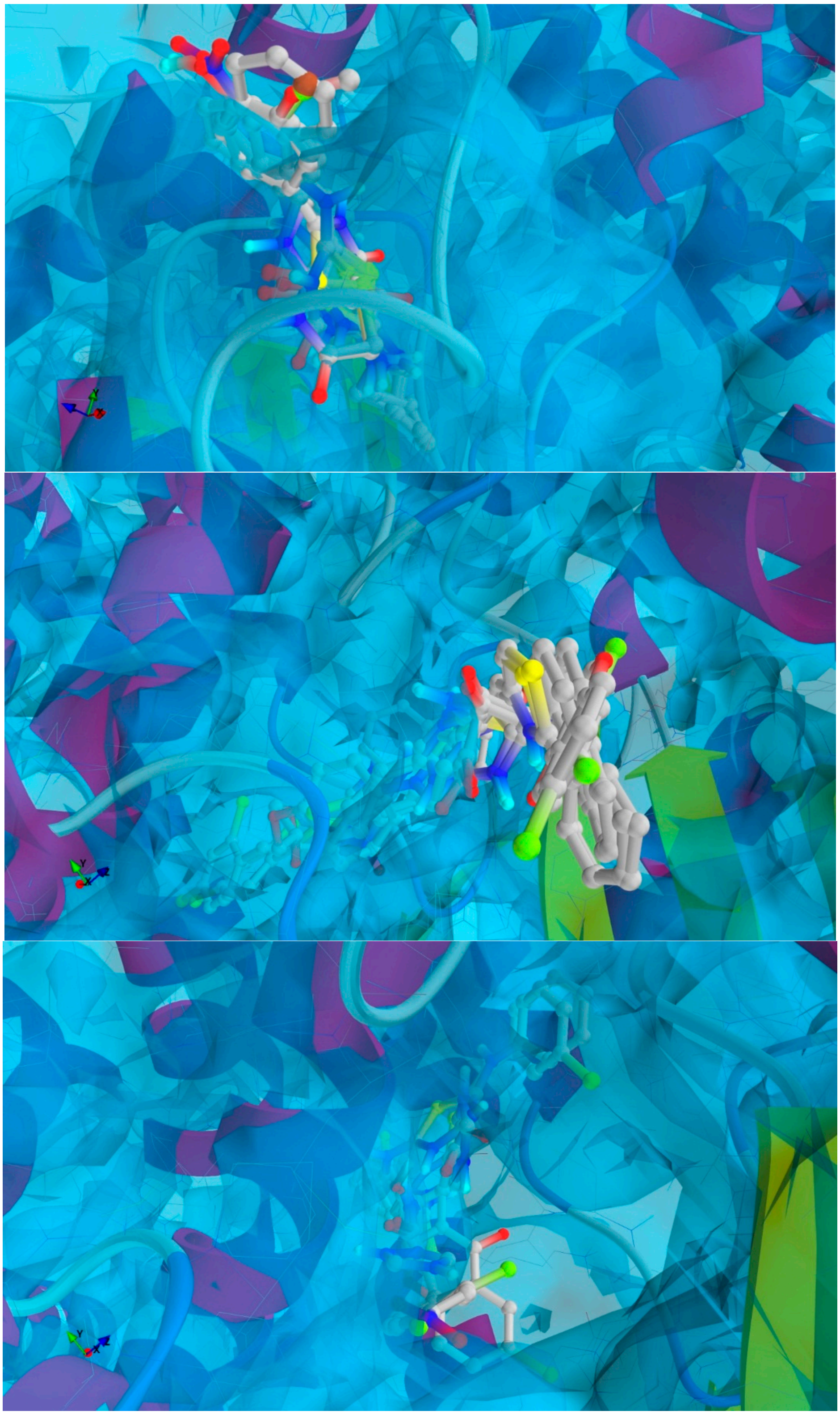

Figure S1. Detailed views of the docking poses of the screened compounds in the active site of lanosterol-14α-demethylase (target is depicted as thin sticks with secondary structure drawn as cartoon backbone with simulation of the molecular surface (semitransparent light blue) for a better understanding of tridimensional positioning in the active site of enzyme, meanwhile ligands are figured as sticks): group A (top image: at the entry of the active site): **2**, **3a**, **3c**, **3f**–**h**, **5**, **8**, **10** and **11**; group B (middle image: at opposite entry of the active site): **3b**, **3d**–**e**, **6d**, **9a**–**9c** and **Ket** (**9b**–**c** and **Ket** are binding in the distal region of this entry); group C (down image: deeply inside of the active site): **6a**–**c**, **6e** and **Flu**.
